# Integrated genomic-metabolic classification of acute myeloid leukemia defines a subgroup with *NPM1* and cohesin/DNA damage mutations

**DOI:** 10.1038/s41375-021-01318-x

**Published:** 2021-06-30

**Authors:** Giorgia Simonetti, Carlo Mengucci, Antonella Padella, Eugenio Fonzi, Gianfranco Picone, Claudio Delpino, Jacopo Nanni, Rossella De Tommaso, Eugenia Franchini, Cristina Papayannidis, Giovanni Marconi, Martina Pazzaglia, Margherita Perricone, Emanuela Scarpi, Maria Chiara Fontana, Samantha Bruno, Michela Tebaldi, Anna Ferrari, Maria Teresa Bochicchio, Andrea Ghelli Luserna Di Rorà, Martina Ghetti, Roberta Napolitano, Annalisa Astolfi, Carmen Baldazzi, Viviana Guadagnuolo, Emanuela Ottaviani, Ilaria Iacobucci, Michele Cavo, Gastone Castellani, Torsten Haferlach, Daniel Remondini, Francesco Capozzi, Giovanni Martinelli

**Affiliations:** 1 Biosciences Laboratory, IRCCS Istituto Romagnolo per lo Studio dei Tumori (IRST) “Dino Amadori”, Meldola, FC Italy; 2 Department of Experimental, Diagnostic and Specialty Medicine, University of Bologna, Bologna, Italy; 3 Department of Agricultural and Food Sciences, University of Bologna, Cesena, FC, Italy; 4 Department of Physics and Astronomy, University of Bologna, Bologna, Italy; 5 Unit of Biostatistics and Clinical Trials, IRCCS Istituto Romagnolo per lo Studio dei Tumori (IRST) “Dino Amadori”, Meldola, FC Italy; 6 Departamento de Ingeniería Química, Universidad Nacional del Sur, Bahía Blanca, Argentina; 7 IRCCS Azienda Ospedaliero-Universitaria di Bologna, Istituto di Ematologia “Seràgnoli”, Bologna, Italy; 8 Hematology Unit, IRCCS Istituto Romagnolo per lo Studio dei Tumori (IRST) “Dino Amadori”, Meldola, FC Italy; 9 Giorgio Prodi” Cancer Research Center, University of Bologna, Bologna and Department of Biomedical and Specialty Surgical Sciences, University of Ferrara, Ferrara, Italy; 10 Department of Pathology, St. Jude Children’s Research Hospital, Memphis, TN USA; 11 MLL Munich Leukemia Laboratory, Munich, Germany

**Keywords:** Cancer genomics, Acute myeloid leukaemia, Cancer metabolism

## Abstract

Although targeting of cell metabolism is a promising therapeutic strategy in acute myeloid leukemia (AML), metabolic dependencies are largely unexplored. We aimed to classify AML patients based on their metabolic landscape and map connections between metabolic and genomic profiles. Combined serum and urine metabolomics improved AML characterization compared with individual biofluid analysis. At intracellular level, AML displayed dysregulated amino acid, nucleotide, lipid, and bioenergetic metabolism. The integration of intracellular and biofluid metabolomics provided a map of alterations in the metabolism of polyamine, purine, keton bodies and polyunsaturated fatty acids and tricarboxylic acid cycle. The intracellular metabolome distinguished three AML clusters, correlating with distinct genomic profiles: *NPM1*-mutated(mut), chromatin/spliceosome-mut and *TP53*-mut/aneuploid AML that were confirmed by biofluid analysis. Interestingly, integrated genomic-metabolic profiles defined two subgroups of *NPM1*-mut AML. One was enriched for mutations in cohesin/DNA damage-related genes (*NPM1*/cohesin-mut AML) and showed increased serum choline + trimethylamine-N-oxide and leucine, higher mutation load, transcriptomic signatures of reduced inflammatory status and better ex-vivo response to EGFR and MET inhibition. The transcriptional differences of enzyme-encoding genes between *NPM1*/cohesin-mut and *NPM1*-mut allowed in silico modeling of intracellular metabolic perturbations. This approach predicted alterations in NAD and purine metabolism in *NPM1*/cohesin-mut AML that suggest potential vulnerabilities, worthy of being therapeutically explored.

## Introduction

Current personalized therapeutic approaches in acute myeloid leukemia (AML) are generally restricted to those patients with identifiable and targeteable genomic lesions [1–3]. However, these approaches do not target interactions between cancer-related features and homeostatic mechanisms that define the leukemic phenotype.

The metabolome is the result of genome- and proteome-wide interactions and is shaped by microenvironmental factors. The biofluid metabolome has been extensively investigated to identify predictive signatures in cardiovascular disorders [4], diverticular disease [5] and diabetes [6], and specific metabolic profiles have been associated with cancer risk [7]. In oncology, metabolomics is a valuable approach for diagnosis, prognostication, and disease monitoring [8]. A paradigmatic example in AML is the accumulation of serum, urine, and intracellular 2-hydroxyglutarate (2-HG) in *IDH1/2*-mutated (mut) cases [9, 10]. 2-HG is an oncometabolite [11, 12], predicts clinical outcome [13], and is a noninvasive biomarker of disease activity [10].

Aberrant enzymatic activity drives cancer metabolic reprogramming and cooperates with mutations of tumor suppressors and oncogenes in pathogenesis. For example, AML cells reduce both host insulin sensitivity and secretion to increase glucose availability for malignant cells [14]. The glycolytic pathway sustains leukemia maintenance and progression. AML cells have a higher mitochondrial (mt) mass and oxygen consumption rate than normal hematopoietic cells [15]. Moreover, leukemia stem cells (LSCs) are addicted to oxidative phosphorylation (OXPHOS) for energy production [16]. OXPHOS is sustained by elevated amino acid metabolism in LSC from de novo AML [17], with cysteine playing a crucial role [18], and is controlled by glutamine levels [19]. Targeted inhibition of these pathways, among others, induces cell death and/or differentiation of AML cells [15, 17–21]. However, the specific response of AML molecular subtypes to agents targeting metabolism has been rarely investigated [22–25].

Here we report an integrated genomic-metabolic study in AML that identified, based on intracellular and the biofluid metabolic profile, a specific *NPM1*-mut AML subgroup characterized by mutations of genes involved in DNA damage response and/or chromatid cohesion (*NPM1*/cohesin-mut) and high levels of serum choline + trimethylamine-N-oxide, and leucine. In silico modeling of the intracellular metabolome based on transcriptomic data highlighted perturbations in the purine and NAD metabolic pathways as *NPM1*/cohesin-mut-specific alterations.

## Materials/subjects and methods

### Metabolomic study design

Participants were included if they were free from infective, autoimmune, celiac, or metabolic diseases such as diabetes and dyslipidaemia. Kidney and liver integrity were also checked. Subjects with acute or chronic renal or hepatic disease, renal or hepatic impairment, cardiovascular disease or a history of neoplasia were excluded from the control cohort. Serum samples from of 119 AML and 145 healthy subjects and urine samples of 103 AML and 139 controls were collected in the fasting state (in the morning). All participants were Caucasian except for 5 (3.4%) healthy controls and six (5%) AML patients. To reduce potential bias and variation unrelated to AML pathogenesis and to ensure that the observed metabolic differences were not due to external confounders, we collected, when possible, two independent serum and urine samples from each patient (more than 50% of cases). Moreover, information on age, gender (the cohorts were balanced for gender), race, health status, diet, drug intake, physical exercise was collected along with specimens and used to filter nuclear magnetic resonance (NMR) spectra during the quality control procedures.

### Nuclear magnetic resonance (NMR) spectroscopy

Serum and urine samples were analyzed by NMR spectroscopy (Supplementary Methods). A stochastic GridSearch was implemented to select the best combination of parameters for dimensionality reduction and classifier performances. Unsupervised and supervised dimensionality reduction were performed using principal component analysis (PCA) and partial least squares discriminant analysis (PLSDA)-sparse(s)PLSDA, respectively. For subset extraction, weights were obtained after signal smoothing via signal-to-noise ratio threshold (which was essential due to unavoidable use of data scalers for dimensionality reduction). The latent components of spectra containing maximum information related to molecular features were identified by a genomic-guided semisupervised approach. This means that the combination of urine and sera latent components used for clustering is extracted with classifiers-derived scores, from classifiers trained with the purpose of discriminating *TP53*-mut/aneuploid, *NPM1*-mut and chromatin/spliceosome-mut samples. Signals in the spectra corresponding to loadings and weights emerging from different tasks were checked for alignment. To minimize the possibility of confounding effects, every step of each classification and clustering task was crossvalidated through suitable k-folds, stratified for gender and age when possible depending on class sizes and sample sizes for the tasks.

### Mass spectrometry (MS)-based metabolomics and data analysis

MS-based meabolomics was performed using an ultra-performance liquid chromatography (Waters ACQUITY, Waters, Milford, MA, USA) and a Q-Exactive high resolution/accurate mass spectrometer (Thermo Fisher Scientific, Waltham, MA, USA) interfaced with a heated electrospray ionization (HESI-II) source and Orbitrap mass analyzer operated at 35.000 mass resolution (Metabolon, Morrisville, NC, USA). Raw data were extracted, peak-identified and QC processed. Compounds were identified by comparison with library entries of purified standards or recurrent unknown entities. Peaks were quantified using area-under-the-curve. Metabolite levels were normalized to DNA content.

### Whole exome sequencing (WES)

WES was performed on 100 AML cases, 17 belonging to a published dataset [26] and 83 new cases. Libraries were prepared from matched tumor and germline DNA (saliva or complete remission samples, Nextera Rapid Capture Expanded and TruSeq Rapid Exome kits, Illumina, San Diego, CA, USA) according to manufacturer’s protocol, and 75/125-bp paired-end sequences were generated (Illumina NextSeq550/HiSeq2500, Illumina). A detailed description is reported in the Supplementary Methods and Tables S1, S2. Sequencing data are available in the European Genome-Phenome Archive (EGAS00001005422).

### Constraint-based metabolic network analysis

We translated gene expression alterations into constraints reducing the feasible space of a metabolic network model (adapted from Shlomi et al. [27]). The impact of a set of these constraints on the feasible space of the metabolic network was evaluated by calculating the minimum and maximum reaction rates (flux variability analysis, FVA), and the instantaneous capability of the network to produce/consume a certain metabolite. Details are reported in the Supplementary Methods. Codes used in constraint-based metabolic network analysis are available in https://github.com/cladelpino/GenePerturbations.

### Statistics

Associations in contingency tables were performed by the Monte Carlo (*B* = 1000,000) simulated Fisher’s exact test. Continuous variables were compared with Mann–Whitney, Kolmogorov–Smirnov, Kruskal–Wallis test, or Welch’s *t*-test. All tests were performed using either python v3.6.5 [28] (packages scipy v1.3.2 [29], statsmodels v0.10.1 [30]) or Rv3.6.3 [31]. When appropriate, *p* values were adjusted for multiple comparisons using the Bonferroni or Benjamini–Hochberg method. To investigate the distribution of sera profile according to blast percentage, samples were divided in three classes (bone marrow: 20–49%, 50–74%, ≥75% blasts, peripheral blood: <30%, 30–69%, ≥70% blasts, according to tertiles). In the drug response analysis, the average area-under-the curve values of the two cohorts were compared. NMR peaks, signal integrals (related to metabolite concentration) and intracellular metabolite levels among three groups were compared by Kruskal–Wallis test. For intracellular metabolite levels Welch *t*-test was also used as post-hoc test. Random Forest analysis was used to estimate the accuracy of individual classification in each group based on metabolomic data. Metabolic pathway analysis was performed using Metaboanalyst (http://www.metaboanalyst.ca) with KEGG annotation. A threshold of five standard deviations from the mean of the control population was used for the identification of outliers.

## Results

### The combined analysis of serum and urine profiles improves AML metabolic characterization

Given that the metabolite composition of biofluids reflects the real-time activity of all biochemical processes in the body and that leukemic cells alter systemic physiology [14], we compared the profile of blood (Table S3) and urine (Table S4) metabolites of AML patients (serum: 88 at diagnosis and 31 at relapse, urine: 80 at diagnosis and 23 at relapse) and healthy controls (CTRL, serum: 145, urine: 139).

**Fig. 1:**
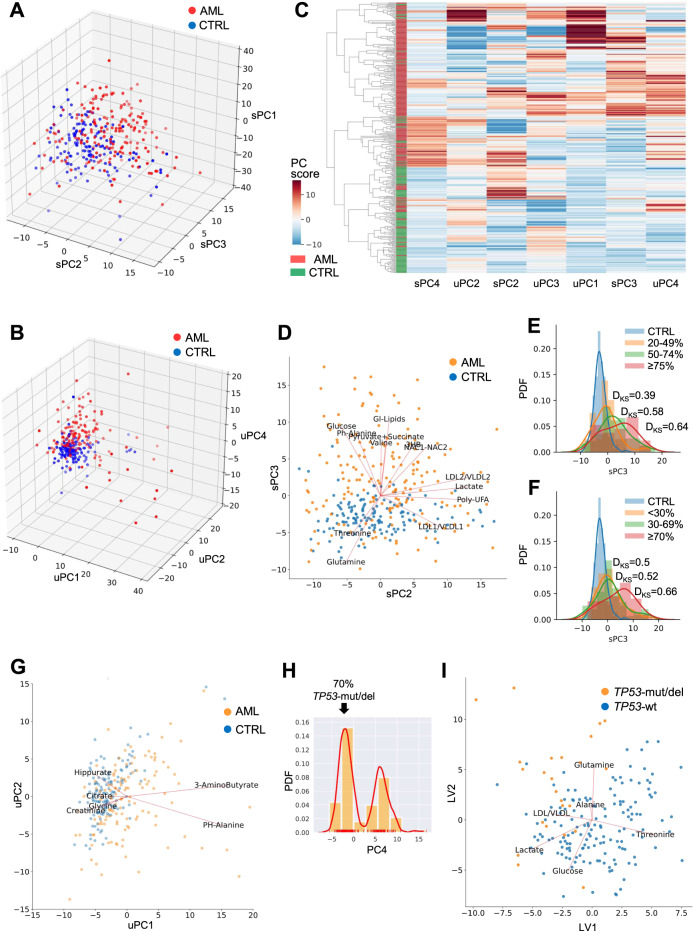
Serum and urine metabolic profile of AML. **A** 3D representation of principal component (PC)1, PC2, and PC3 projection of serum NMR data of AML and healthy controls (CTRL), which accounted for 53% of the total explained variance. **B** 3D representation of PC1, PC2, and PC4 projections of urine NMR data of AML and healthy controls. **C** Hierachical clustering of AML and controls using integrated serum (*n* = 3) and urine (*n* = 4) PCs, selected as the best combination of predictive features by comparing an AdaBoost Classifier and a SVM Classifier. The integration yielded an enhanced coherence in adjacency between AML and controls compared with single biofluid analysis. Each component contains linear combinations of signature metabolites shown in biplots for both sera and urine samples. Colors indicate the score on each PC. **D** BiPlot on PCA reduced space of serum NMR data. Metabolites showing significant alterations (*p* < 0.05) were plotted along their maximum variance direction in the PCA score space. Only completely template-matched signals were reported. **E** Estimated probability density functions (PDFs) of serum PC3 scores of AML cases according to bone marrow blast percentage (20–49%: *p* = 1.66e−04; 50–74%: *p* = 6.47e−10; ≥75%: *p* = 1.67e−15) and **F** peripheral blood blast percentage (<30%: *p* = 8.85e−08; 30–69%: *p* = 5.98e−10; ≥70%: *p* = 8.33e−15). The similarity between each AML blast count class and CTRL was computed using the score distribution of serum PC3, which is the latent variable best separating AML and CTRL in the metabolic latent space (D_KS_: absolute value of the maximal difference between the cumulative function of two distributions, representing the maximal distance between them, according to Kolmogorov–Smirnov statistics). **G** BiPlot on PCA reduced space of urine NMR data. Metabolites were plotted as in (**D**). **H** Serum PC4 scores in AML patients (median value: group 1, −1.94 and group 2, 6.35). **I** BiPlot on PLSDA reduced space (from a 5-PLSDA-component AdaBoost classification) for *TP53*-wt and *TP53*-mut/del AML. Metabolites were plotted along their maximum variance direction in the PLSDA score space (LV latent variables).

The metabolomic profile provided efficient discrimination between patient and CTRL both at serum and urine level, with an accuracy of 83% (Figs. 1A and S1) and 85% (Figs. 1B and S2), respectively. Since patient and CTRL cohorts were not age-matched (median age: AML, 67-years (18–90), CTRL, 57-years (23–75)), we verified that age had no significant effects on the classification (Table S5). Notably, the integration of serum and urine data yielded an average accuracy of 90% in the separation of AML and CTRL (Fig. 1C), by using a reduced number of features (Fig. S3) compared with the analysis of each biofluid per se. In serum, PC2–3 space gave the best 2D combination for AML-CTRL separation, with 13 metabolites showing signficantly different levels (*p* < 0.05, Fig. 1D and Table 1). These metabolites were not significantly correlated with age or gender (Table S6). Amino acid and tricarboxylic acid cycle (TCA) cycle byproducts, that had increased concentration in AML except for glutamine and threonine, mainly represented variance in PC3, while lactate and fatty acid metabolism compounds accounted for variance in PC2 (Fig. 1D). When looking at sample distribution along serum PC3, that provided a good discrimination between AML and CTRL, we observed that all AML subgroups were significantly different from normal cases, independently of bone marrow or peripheral blast percentage (Fig. 1E, F). Moreover, a low bone marrow blast percentage (20–49%, Fig. 1E) and a high peripheral blood blast percentage (≥75%, Fig. 1F) resulted in a reduced and increased distance from CTRL, respectively.

**Table 1:** Metabolic alterations in serum and urine samples from AML patients compared with those from controls.

Metabolite	Biplot name	Changes in AML vs. CNTRL	Biofluid	Kruskal–Wallis *p* -value
3-Hydroxybutyrate	3HB	↑	Serum	<0.001
Glycerol of lipids	Gl-Lipids	↑	Serum	<0.001
Glucose	Glucose	↑	Serum	0.015
Glutamine	Glutamine	↓	Serum	<0.001
Lactate	Lactate	↑	Serum	0.033
Low density/very low density lipids1	LDL1/VLDL1	↓	Serum	<0.001
Low density/very low density lipids2	LDL2/VLDL2	↑	Serum	0.019
N-acetylglycoproteins (1 and 2)	NAC1/NAC2	↑	Serum	<0.001
Polyunsaturated fatty acids	Poly-UFA	↑	Serum	0.008
Pyruvate + Succinate	Pyruvate + Succinate	↑	Serum	0.033
Threonine	Threonine	↓	Serum	<0.001
Valine	Valine	↑	Serum	0.022
Phenylalanine	Ph-Alanine	↑	Serum/urine	0.008/<0.001
3-Aminobutyrate	3-Aminobutyrate	↑	Urine	0.006
Citrate	Citrate	↓	Urine	<0.001
Creatinine	Creatinine	↓	Urine	<0.001
Glycine	Glycine	↓	Urine	0.007
Hippurate	Hippurate	↓	Urine	<0.001

↑: up; ↓: down.

Moreover, we detected increased concentration of 3-aminobutyrate and phenylalanine in the urine of AML patients compared with CTRL (Fig. 1G, Tables 1 and S6). Citrate, creatinine, and hippurate, which are among the most abundant urine components, showed low levels in AML, suggesting reduced excretion. Similarly, decreased glycine was indicative of reduced catabolism.

Notably, two groups of patients were distinguished by serum metabolites in PC4 (*p* < 0.001), and one of them included 70% of *TP53*-mut/deleted(del) AML (Fig. 1H). When comparing *TP53*-mut/del and wild-type (wt) AML, we found lower levels of threonine and glucose in *TP53*-mut/del cases (Fig. 1I), that suggested an increased cellular uptake, likely aimed at satisfying macromolecule biosynthesis and bioenergetic requirements [32], with reduced lactate excretion [33].

Overall, integration of serum and urine metabolomics improved the prediction accuracy with respect to single biofluid classification.

### CD34^+^ and CD33^+^ AML cells have dysregulated lipid, amino acid, nucleotide, and bioenergetic metabolism

**Fig. 2:**
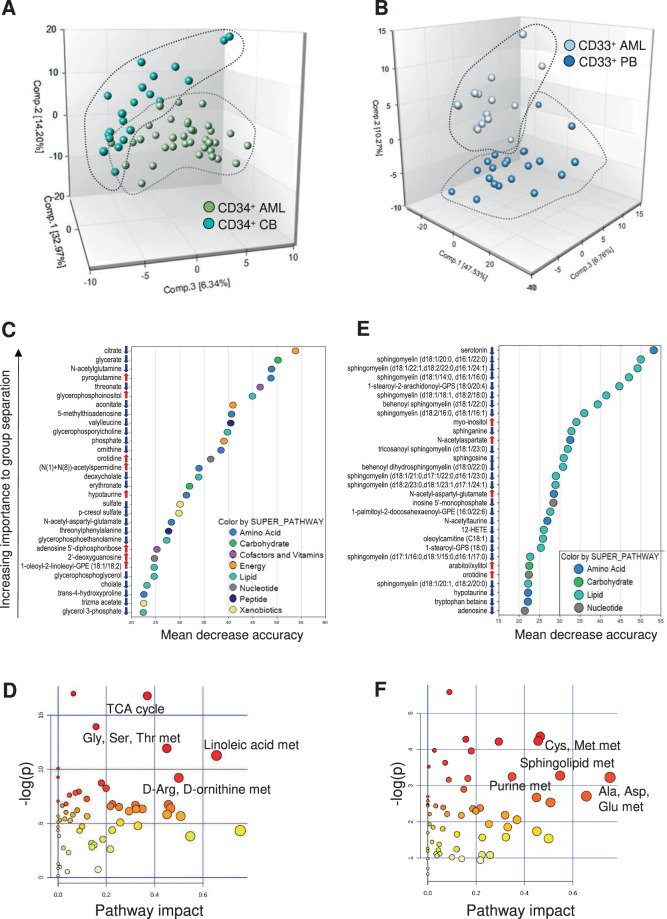
Intracellular metabolomics of AML. PCA of the metabolic profile of **A** CD34^+^ and **B** CD33^+^ AML cells compared to their healthy control populations (CD34^+^ CB and CD33^+^ PB cells). **C** Biochemical importance plot of the top 30 metabolites contributing to group separation between CD34^+^ AML and CD34^+^ CB stem-progenitor cells. Red and blue arrows indicate increased or decreased metabolite levels in AML cells compared with CTRL cells (|fold change | ≥ 2, *q* ≤ 0.05), respectively. **D** Altered metabolic pathways in CD34^+^ AML cells. The most significant pathways with the strongest impact on CD34^+^ AML cells are shown. **E** Biochemical importance plot of the top 30 metabolites contributing to group separation between CD33^+^ AML blasts and CD33^+^ PB cells from CTRL (red and blue arrows as in (**C**)). **F** Altered metabolic pathways in CD33^+^ AML cells. The most significant pathways with the strongest impact on CD33^+^ AML cells are shown.

To obtain a metabolic fingerprint of AML, we performed intracellular metabolic profiling of leukemic cells (35 CD34^+^ and 15 CD33^+^ isolated bone marrow (BM) blasts) and compared them with 21 normal cord blood (CB) CD34^+^ and 21 normal CD33^+^ peripheral blood (PB) samples from healthy subjects. CD34^+^ AML and CD33^+^ AML segregated from their normal counterparts (Fig. 2A, B), with a predictive accuracy of 85.7% and 94.4%, respectively (Fig. S4A, B), but not from each other (Fig. S4C). Among the 300 detected metabolites, 66 and 35 were down- and upregulated, respectively, in CD34^+^ AML cells, while 102 and 19 showed reduced and increased levels, respectively, in CD33^+^ AML compared with their control group. No significant differences in metabolite levels were detected between CD34^+^ and CD33^+^ AML.

The top scored 30 biochemicals that distinguished CD34^+^ AML from CD34^+^ CB cells were primarily involved in bioenergetics, amino acid, and lipid metabolism (Fig. 2C). Overall, 41 pathways were dysregulated in CD34^+^ AML, with TCA cycle, D-Arginine and D-ornithine and linoleic acid metabolism showing the strongest impact (Fig. 2D and Table S7).

When comparing CD33^+^ AML and CD33^+^ PB, the top discriminating 30 biochemicals included lipids, nucleotides, and amino acid metabolism (Fig. 2E), with alanine, aspartate and glutamate, cysteine and methionine, purine and sphingolipid metabolic pathways showing the strongest impact (Fig. 2F and Table S8).

Lipid, amino acid, nucleotide, and bioenergetic metabolism were confirmed as the most widely altered pathways when comparing the whole AML and CTRL cohorts (Fig. S4D and Table S9; 17 increased and 147 decreased metabolites), which were separated with a predictive accuracy of 89.1% (Fig. S4E).

### Integrated intracellular and biofluid metabolomics highlighted alterations in the metabolism of polyamine, purine, keton bodies and polyunsaturated fatty acids and in the TCA cycle in AML

**Fig. 3:**
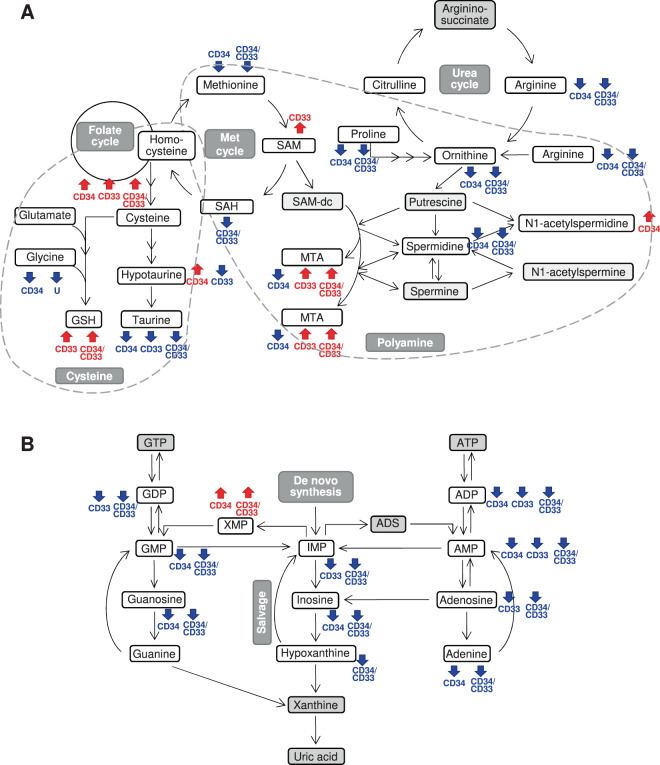
Schematic representation of polyamine, cysteine, and purine metabolic pathways integrating intracellular and biofluid metabolomic data. **A** Polyamine and cysteine metabolic pathway and urea cycle. **B** Purine metabolism. Red and blue arrows/text indicate increased or decreased metabolite levels in AML cells compared with their CTRL, respectively (|fold change | ≥2, *q* ≤ 0.05) and in the urine of patients compared with CTRL (*p* < 0.05). Gray metabolite boxes indicate the ones that were not detected by MS analysis. The schemes report the most relevant metabolites in the pathway according to metabolomic data (ADP adenosine 5′-diphosphate, ADS adenosine, AMP adenosine 5′-monophosphate, ATP adenosine 5′-triphosphate, dcSAM decarboxylated S-adenosylmethionine, GDP guanosine 5′-diphosphate, GMP guanosine 5′-monophosphate, GTP guanosine 5′-triphosphate, GSH reduced glutathione, IMP inosine 5′-monophosphate, MET methionine, MTA 5-methylthioadenosine, SAH S-adenosylhomocysteine, SAM S-adenosylmethionine, XMP xanthosine 5′-monophosphate).

**Fig. 4:**
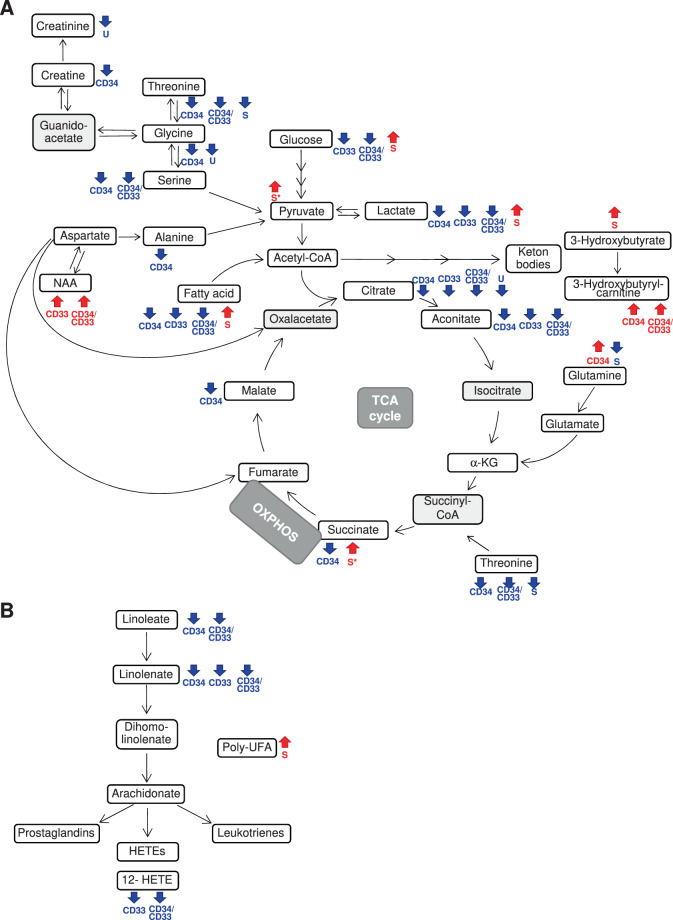
Summary of AML metabolic alterations in the TCA cycle, linoleic acid metabolism, and related pathways. **A** TCA cycle and related amino acid pathways. **B** Linoleic acid metabolism. Red and blue arrows/text indicate increased or decreased metabolite levels in AML cells versus their CTRL, respectively (|fold change | ≥ 2, *q* ≤ 0.05) and in the serum or urine of patients compared with CTRL (*p* < 0.05). Gray metabolite boxes indicate the ones that were not detected by MS analysis. The schemes report the most relevant metabolites in the pathway according to metabolomic data (HETE hydroxyeicosatetraenoic acid, NAA N-acetylaspartate, poly-UFA polyunsaturated fatty acids, TCA trycarboxylic acid cycle).

After showing a distinct metabolomic profile for leukemic compared to normal CD34^+^ or CD33^+^ cells, we next focused on the significantly dysregulated metabolic pathways. We observed decreased arginine, methionine, and proline in leukemic cells, that suggested elevated polyamine biosynthesis (S-adenosylmethionine, 5-methylthioadenosine, and N1-acetylspermidine in CD33^+^ and CD34^*+*^ cells, respectively, Fig. 3A), which in turn supports cell proliferation [34]. Accordingly, the low levels of purine nucleotides (Fig. 3B) may indicate enhanced production of adenosine 5′-triphosphate and guanosine 5′-triphosphate that are crucial for providing cellular energy and intracellular signaling, respectively [35]. Tumor growth was also supported by elevated N-acetylaspartate levels in leukemic cells (Fig. 4A) [36]. Of note, in the CD33^+^ cohort, *NPM1*-mut AML scored as outliers for their high levels of the N-acetylaspartate derivative N-acetyl-aspartyl-glutamate (90.9% and 25.0% of *NPM1*-mut AML among outliers and non-outliers, respectively, *p* = 0.033). Moreover, high levels of reduced glutathione (Fig. 3A) and ophtalmate (data not shown) were indicative of elevated cellular oxidative stress, and increased 3-hydroxybutyrate in the serum of patients and of 3-hydroxybutyrylcarnitine in leukemic cells reflected heightened ketogenesis in AML (Fig. 4A and Table S10). Polyunsaturated fatty acids (Fig. 4B) and glucose (Fig. 4A) were elevated in the serum of patients but reduced in CD33^+^ and/or CD34^+^ leukemic cells compared with normal ones, suggesting the need for a constant energy reservoir that is rapidly consumed by cells. The reduced levels of intracellular TCA intermediates and of serum glutamine were also indicative of increased bioenergetics requirement, especially in the CD34 compartment (Fig. 4A). This requirement was further supported by decreased levels of amino acid sources of pyruvate (e.g. threonine, glycine, serine, alanine), with a significant increase of serum lactate, an end-product of glycolysis and glutaminolysis (Fig. 4A). In parallel, intracellular lactate levels were lower in both CD34^+^ and CD33^+^ AML than normal cells, thus suggesting a high excretion capacity (Table S10).

### Metabolic clusters define AML subgroups with different genomic features

**Fig. 5:**
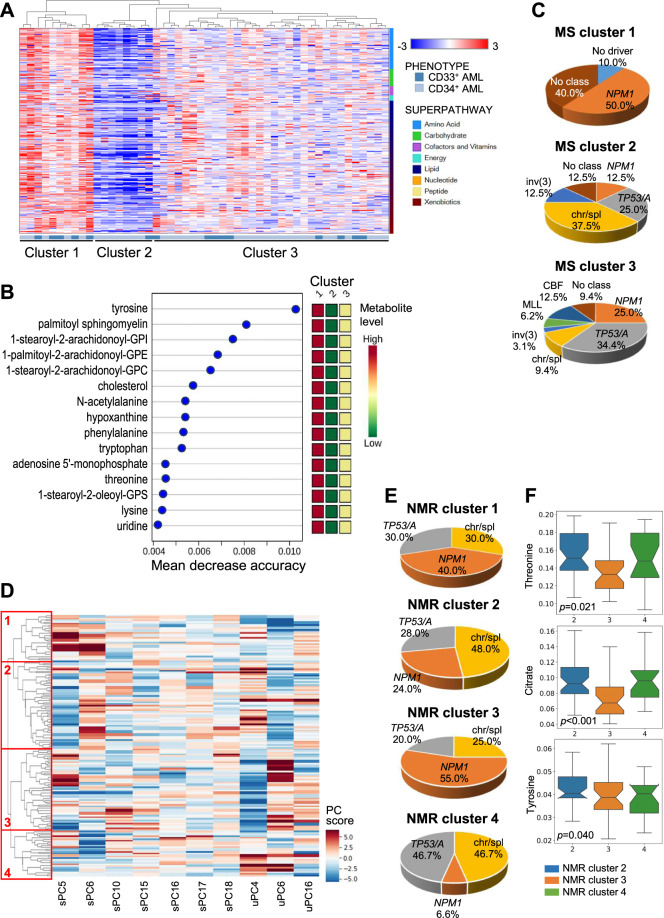
Intracellular and biofluid metabolomics show association with AML molecular classification. **A** Unsupervised hierarchical clustering of AML according to intracellular metabolomic profiles (MS, each row denotes a metabolite, each column a sample). **B** Top 15 metabolites contributing to separation of the three MS metabolic clusters (1, 2, 3). The metabolites belong to the following superpathways: amino acids and their derivatives (tyrosine, N-acetylalanine, phenilalanine, tryptophan, threonine, lysine), intermediates of purine and pyrimidine metabolism (hypoxanthine, adenosyne-5′-monophosphate, uridine) and lipids (sphingolipid, phosphatidylinositol, phosphatidylethanolamine, phosphatidylcholine, cholesterol, phosphatidylserine. Colored squares on the right indicate metabolite levels in each cluster. **C** Molecular classification of MS metabolic clusters [3]. Due to the low number of t(8;21) and inv(16)/t(16;16) cases, they were grouped in the core-binding factor (CBF) category and a t(6;9) patient with complex karyotype was included in the *TP53*/aneuploidy category (*NPM1* *NPM1*-mut, chr/spl chromatin/spliceosome-mut, *TP53*/A *TP53-*mut/aneuploidy, inv(3) inv(3)/t(3;3), *KMT2A* *KMT2A-*rearranged). **D** Hierarchical clustering of AML patients belonging to the *NPM1*-mut, chromatin/spliceosome-mut or TP53/aneuploidy molecular classes according to biofluid metabolomic profile (NMR). These components were selected as the combination of urine and serum spectral features that best described the above mentioned genomic stratification. Of the ten features selected via stochastic gridsearch, seven came from serum spectra, indicating serum as the principal vector of information for this particular stratification. Colors indicate the score on each PC. **E** Molecular classification of NMR metabolic clusters. **F** Top scoring serum metabolites separating NMR clusters 2, 3, and 4. Signature metabolites were extracted from sera samples by selecting the highest scoring signals in terms of presence amongst the sera PC responsible for the best separation of molecular subgroups and the average of absolute values of their loadings. Statistical significance was obtained with SciPy.Stats Kruskal–Wallis *H*-test using stepdown Sidak correction. Notch width corresponds to the confidence interval of the median.

We then classified AML cases according to their intracellular metabolic profile. Unsupervised hierarchical clustering clearly defined 3 clusters (Fig. 5A and Table S11). The top 15 metabolites that better distinguished the 3 clusters included amino acids and their derivatives (e.g. tyrosine, phenylalanine, tryptophan, threonine, lysine), intermediates of purine and pyrimidine metabolism (e.g. hypoxanthine, adenosine 5′-monophosphate, uridine) and lipids (e.g. palmitoyl sphingomyelin, cholesterol), that showed high, intermediate and low levels in cluster 1, 2 and 3, respectively (Fig. 5B). In order to integrate genomics (Table S12) and metabolomics, we assigned each sample to a molecular class [3]. Cluster-1 was enriched for *NPM1*-mut AML (50.0%), cluster-2 for cases with altered chromatin/spliceosome genes (37.5%), and cluster-3 for *TP53*-mut/aneuploid AML (34.4%, *p* = 0.023, Fig. 5C). We then investigated differences at serum and urine level across genetic categories (chromatin/spliceosome-mut, *NPM1*-mut, *TP53*-mut/aneuploid AML, *n* = 71) and identified 4 NMR clusters (Fig. 5D). Genomic categories associated with specific biofluid metabolic cluster (clusters 2, 3, and 4, *p* = 0.040, Fig. 5E), in accordance with the intracellular metabolic profiles.

High levels of serum tyrosine, threonine, and citrate correlated with the cluster enriched for chromatin/spliceosome-mut. Viceversa, low levels of these metabolites were detected in the cluster enriched for *NPM1*-mut (Fig. 5F). The cluster associated with *TP53*-mut/aneuploid AML displayed intermediate threonine and tyrosine levels and high citrate in the serum compared to the other two clusters. Notably, tyrosine and threonine showed high intracellular levels in the *NPM1*-mut enriched cluster compared with the other clusters (mean decrease accuracy = 0.010 and 0.005, respectively, Fig. 5B), suggesting an increased intracellular need and/or uptake leading to serum depletion.

### NMR-driven metabolic classification identifies two subgroups of *NPM1*-mut patients

**Fig. 6:**
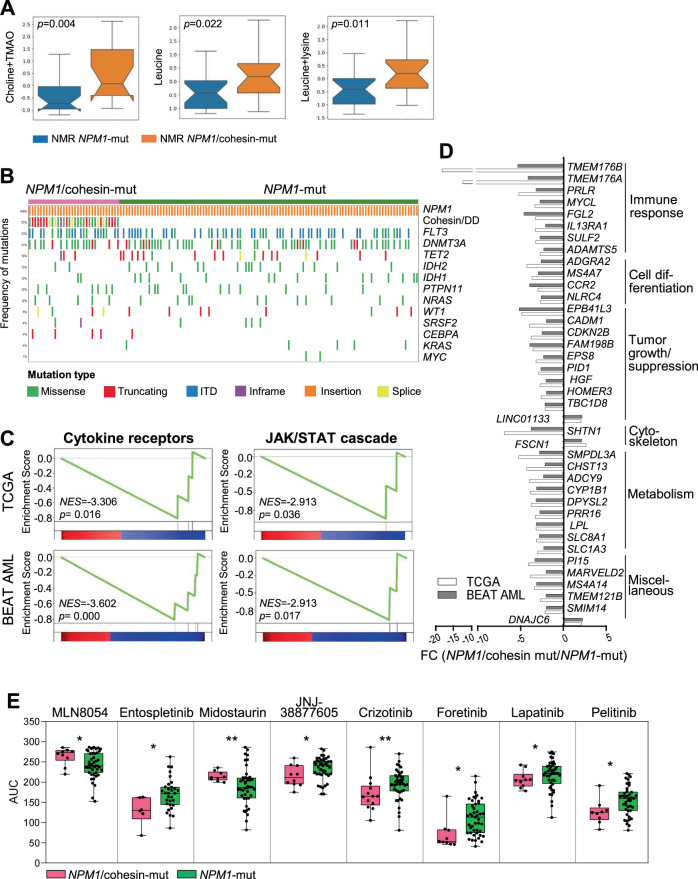
Metabolic, genomic, transcriptomic and drug response differences between *NPM1*/cohesin-mut and *NPM*1-mut AML. **A** Serum metabolites separating *NPM1*/cohesin-mut and *NPM1*-mut AML (TMAO trimethylamine-N-oxide). **B** Oncoprint of mutations in AML-related genes (frequency >3% in the overall population) in *NPM1*/cohesin-mut and *NPM1*-mut AML. WES data were obtained from the TCGA (*n* = 13 *NPM1*/cohesin-mut, *n* = 33 *NPM1*-mut) and BEAT AML (*n* = 19 *NPM1*/cohesin-mut, *n* = 72 *NPM1*-mut, including 7 relapse cases) cohorts. Rows denote genes or groups of genes (cohesin/DD cohesion/DNA damage-related genes). Columns represent frequency of mutations and single patients (ITD internal tandem duplication). **C** Signatures of cytokine receptors and JAK-STAT cascade from GSEA showing significance in both datasets (TCGA, left to right: cytokine–cytokine receptor binding, regulation of JAK-STAT cascade, *n* = 9 *NPM1*/cohesin-mut, *n* = 25 *NPM1*-mut; BEAT AML, left to right: cytokine receptor activity, JAK/STAT cascade, *n* = 14 *NPM1*/cohesin-mut, *n* = 47 *NPM1*-mut, including 3 relapse cases). **D** Genes involved in immune response, cell differentiation, tumor growth regulation, cytoskeleton, metabolism and other cellular processes, showing a significantly different expression between *NPM1*/cohesin-mut and *NPM1*-mut AML in both cohorts. **E** Area under the curve (AUC) for the drugs showing a significantly different response between *NPM1*/cohesin-mut and *NPM1*-mut AML was plotted for the two cohorts (*NPM1*/cohesin-mut, *n* = 6–13; *NPM1*-mut, *n* = 31–45) [1]: MLN8054 (Aurora kinase A inhibitor), Entospletinib (SYK inhibitor), Midostaurin (FLT3, JAK inhibitor), JNJ-38877605 (MET inhibitor), Crizotinib (ALK, MET, ROS1, NTRK inhibitor), Foretinib (MET, KDR, TIE inhibitor), Lapatinib (ErbB-2, EGFR inhibitor), Pelitinib (EGFR inhibitor). Boxes represent the mean (horizontal line) and extend from the 25^th^ to 75^th^ percentiles; whiskers extend from the minimum to the maximum value and each value is plotted (*p ≤ 0.05, **p ≤ 0.01).

Our data so far described a significant association between genomic and metabolic profiles. However, even within the same genomic category, different subgroups can be identified according to combinatorial mutation patterns and consequently they may show metabolic differences. This hypothesis was confirmed in patients carrying *NPM1* mutations, in whom the metabolic profiles defined two distinct subgroups. *NPM1*-mut patients with higher serum levels of choline + trimethylamine N-oxide, leucine and leucine + lysine (*p* < 0.05, Fig. 6A) were enriched for co-occurring mutations in cohesin complex and DNA damage genes (*SMC1A, SMC3, RAD21, STAG2, ATM, ATR, BRCA2*, named *NPM1/*cohesin-mut), compared with *NPM1*-mut patients from the other metabolic group (60.0% versus 9.1% of cases, respectively, *p* = 0.024).

In order to gain insights into molecular mechanisms associated with the metabolic differences between *NPM1*/cohesion-mut and *NPM1*-mut AML, we analyzed paired exome and transcriptome (Fig. S5) data from the TCGA and BEAT AML datasets for the same genetic subgroups. Twenty-three percent of *NPM1*-mut AML (32/137) also carried at least one alteration in recurrently mutated genes [1] belonging to the cohesin complex or DNA damage pathways. Compared with *NPM1*-mut AML, *NPM1*/cohesin-mut cases were characterized by a lower white blood cell count (39.7 vs. 64.1 cells/mm^3^, *p* = 0.006) and a significantly higher mutation load (average mutation number: 15 vs. 9, *p* < 0.001), with lower frequency of *IDH1–2*/*TET2* mutations (21.9% vs. 46.7% of *NPM1*-mut, *p* = 0.014, Fig. 6B). *FLT3* alterations were evenly distributed between the two groups (Fig. 6B) and no differences were observed in clinical outcome (Fig. S6A).

At transcriptional level, signatures of cellular response to cytokines and JAK-STAT cascade were significantly downregulated in *NPM1*/cohesin-mut AML (Fig. 6C). Accordingly, *NPM1*/cohesin-mut AML showed reduced expression of genes involved in the regulation of immune and inflammatory response, along with others related to cell differentiation and metabolism (Fig. 6D).

We then compared the ex vivo response of *NPM1*/cohesin-mut and *NPM1-*mut AML to a panel of targeted agents (*n* = 122, BEAT AML [1]). *NPM1*/cohesin-mut AML showed decreased sensitivity to the Aurora kinase A inhibitor MLN8054 and the FLT3/JAK inhibitor Midostaurin but responded better to SYK, MET, and EGFR inhibitors (Entospletinib, JNJ-38877605, Crizotinib, Foretinib, Lapatinib, Pelitinib, Fig. 6E).

These data suggest that the co-occurrence of different mutations with altered *NPM1* may confer a distinct metabolic, transcriptomic, and drug sensitivity profile to the leukemic cells.

### Predicting metabolic specificities of *NPM1*/cohesin-mut AML

**Fig. 7:**
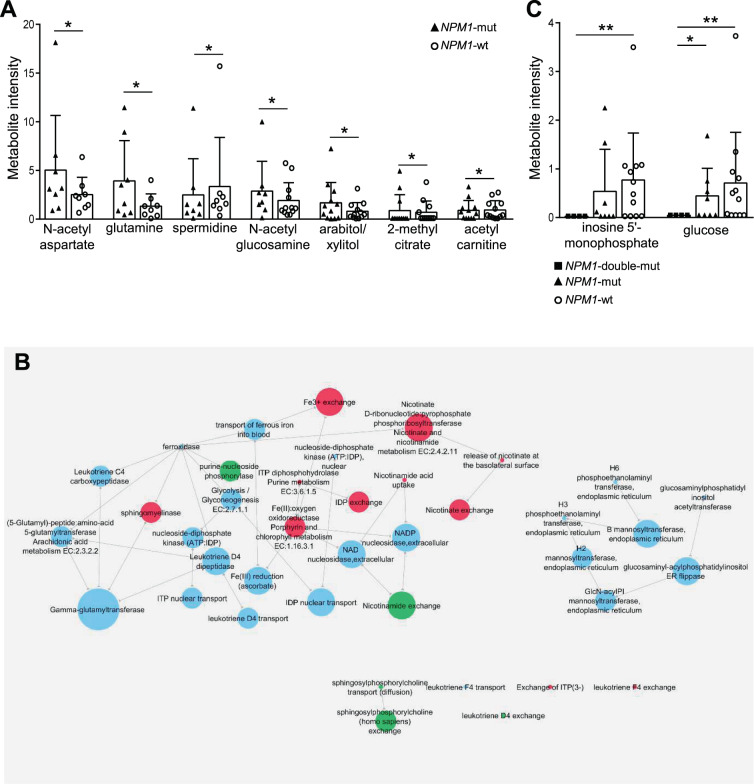
Modeling the metabolic network of *NPM1*/cohesin-mut AML. **A** Intracellular metabolites from FVA analysis showing a significant different intensity between *NPM1*-mut (*n* = 8) and *NPM1*-wt (*n* = 12, normal karyotype) in MS analysis. Metabolite intensity normalized on DNA concentration is shown in the plot. Significance was obtained by Welch *t*-test (*p ≤ 0.05). **B** *NPM1*/cohesin-mut specific metabolic reaction perturbation network. (red: minimum flux, green: maximum flux, light blue: no information among *NPM1*/cohesin-mut-specific alterations). Sizes of nodes are proportional to links originating from that node and pointing towards others (*outdegree)*. **C** Intracellular metabolite intensity of *NPM1*/cohesin-mut-specific perturbations predicted by FVA (*NPM1*/cohesin-mut, *n* = 4; *NPM1*-mut, *n* = 8, *NPM1*-wt, *n* = 12, normal karyotype). Metabolite intensity normalized on DNA concentration is shown in the plot. Significance was obtained by Welch *t*-test (*p ≤ 0.05, **p ≤ 0.01).

Seven downregulated genes in *NPM1*/cohesin-mut compared with *NPM1*-mut AML encoded for enzymes involved in nucleotide (*ADCY9, DPYSL2*), lipid (*LPL*) and carbohydrate (*CHST13*) metabolism, energy production (*CYP1B1*) and transporter/exchanger (*SLC8A1*, *SLC1A3*, Figs. 7C and S6B). We thus modeled the consequences of gene expression alterations of *NPM1*/cohesin-mut AML on the intracellular metabolome by reconstructing genome scale metabolic network models. Based on the analysis of diverse cellular models (Fig. S7A) and our MS data (Fig. S7B), we selected a hematopoietic model derived from Recon2. The selected reconstruction was validated by modeling the effect of *IDH* mutations (Table S13 and Fig. S7C, D).

We first predicted the changes in metabolic fluxes (Table S14) induced by the altered expression of enzymes between *NPM1*-mut and *NPM1*-wt AML (transcriptomic data from the TCGA and BEAT AML cohorts, Fig. S8). Interestingly, among the perturbed metabolites, experimental evidence confirmed increased N-acetylaspartate and glutamine, reduced spermidine levels (among others) in *NPM1*-mut compared with *NPM1*-wt AML (Fig. 7A). We then simulated the intracellular metabolome of *NPM1*/cohesin-mut AML by adding the 7 downregulated genes to the model (Table S15). Eleven metabolites and 42 reactions were predicted to be specifically perturbed in the *NPM1*/cohesin-mut model (Table S16). A metabolic network reconstruction of the altered reactions showed a major cluster centered on nicotinate, nicotinamide, and inosine exchange/modification, with connections to glycolysis and metabolism of leukotriene inflammatory molecules (Fig. 7B), that were also confirmed by pathway enrichment analysis of genes catalyzing the network reactions (Fig. S9 and Table S17). Notably, *NPM1*/cohesin-mut AML showed lower intracellular levels of inosine-5′-monophosphate and glucose when compared with *NPM1*-wt and/or *NPM1*-mut AML (Fig. 7C). Overall, our multistep approach defined the metabolic specificities of *NPM1*/cohesin-mut AML.

## Discussion

Few studies have previously analyzed the metabolic profile of AML patient serum [37–41] or of a limited number of primary cells [17, 42, 43]. Here, we have performed integrated genomics and metabolomics analysis in AML, which showed genetic-related differences in the metabolic profiles and defined multiple subgroups with distinct constellations of mutations and metabolic features.

Among the endogenous factors influencing the human metabolome, age and sex have a strong influence, at least in healthy subjects [44]. However, we can exclude confounding effects in our results for the following reasons: (i) the majority of metabolic alterations occurred in the opposite direction to the one expected as a readout of confounding factors [44]; (ii) the correlation coefficient for the identified metabolites with age and gender was not significant; (iii) some of the data obtained on serum were also reported in previous studies [37–41].

First, integrated serum and urine analysis accurately discriminated between AML and normal patients, suggesting a robust approach for evaluating disease metabolic subgroups and a valid, low-cost approach for noninvasive population analyses.

Second, we integrated biofluid and intracellular metabolomics. We used NMR and MS as complementary techniques for biofluids and primary cell profiling, respectively. The rationale of this approach is twofold: it allowed us to benefit from the reproducibility of NMR, which offers unbiased information and could enable a rapid translation to the clinical practice, and from the high sensitivity of MS in metabolite detection from low cell numbers. Our comprehensive view showed alterations in the TCA cycle and in the metabolism of purine nucleotides, amino acid, fatty acids, keton bodies, polyamine, glutamine and other amino acids. The incomplete overlap between the metabolic alterations observed when comparing CD34^+^ and CD33^+^ blasts with their respective healthy populations may be partly due to the usage of CD34^+^ CB and CD33^+^ PB cells, as controls. Notably, many of the identified pathways can be therapeutically exploited (e.g. glutaminolysis, arginine uptake, aspartate production, fatty acid oxidation, polyamine metabolism, ketogenesis) and the inhibition of some of them achieved promising results in AML [17, 45] or in cancer [46, 47] models.

When integrated with genetic features, the metabolic profiles showed association with *NPM1*-mut, chromatin/spliceosome-mut and *TP53*-mut/aneuploid AML classes. Chromatin/spliceosome-mut and *TP53*-mut/aneuploid AML shared some metabolic features, according to their clustering. This may be partly explained by the recurrence of aneuploidies in the chromatin/spliceosome-mut class (42.9% of those classified in the *TP53*-mut/aneuploid AML associated cluster). *NPM1*-mut AML showed high intracellular levels of N-acetyl-aspartyl-glutamate, that have been previously associated with *MYC* activation [48]. Indeed, mutant *NPM1* indirectly stabilizes c-MYC protein [49] and an oncogenic *MYC* mutation was also detected in a *NPM1*-mut AML case, in line with previous findings [3].

Our data also classified *NPM1*-mut AML carrying mutations in cohesin or DNA damage-related genes as a distinct metabolic subgroup. This group does not associate with *IDH1–2*/*TET2* mutations, which are also frequently observed in *NPM1*-mut cases [1, 2, 50] but it was characterized by higher mutation burden, lower white blood cell count and dowregulation of immune-related genes [51]. Accordingly, in silico modeling of the *NPM1*/cohesin-mut-specific metabolic perturbations predicted changes in the balance of leukotrienes. Among the metabolic genes downregulated in *NPM1*/cohesin-mut compared with *NPM1*-mut AML, *CHST13*, *ADCY9*, *PRR16*, *LPL*, and *SLC1A3* were confirmed by *STAG2*-deficient model of *NPM1*-mut leukemia [52]. Moreover, flux and network analysis based on the identified transcriptomic changes pointed at alterations in the purine and NAD superpathways as *NPM1*/cohesin-mut-specific ones. Inosine-5′-monosphate, an intermediate in the purine metabolism, showed low levels in *NPM1*/cohesin-mut compared with *NPM1*-mut AML cells. Of note, inosine favors spontaneous mutations, since a base transition is introduced when it is incorporated into newly synthesized DNA [53]. In addition, both purines and NAD regulate immune function and cytokine release [54, 55]. Given that NAD modulates gene expression [56] and that cohesin gene mutations alter chromatin accessibility [57], the targeting of NAD metabolism could restore the myeloid differentiation program in *NPM1*/cohesin-mut leukemic cells. Future studies taking into account leukemic cell metabolism and microenvironmental factors, will further investigate the suggested vulnerabilities. With regard to therapeutics, *NPM1*/cohesin-mut AML were more sensitive than *NPM1*-mut AML to EGFR inhibition, which may lead to the release of the differentiation brake [58] and to drugs targeting the tyrosine kinase receptor MET, likely due to a mild autocrine pathway activation in these cases, who express low levels of the ligand [59].

Overall, our results provide a map of the crosstalk between metabolic pathways and between genomics and metabolomics in AML, reflecting functional interactions and dependencies that could be therapeutically exploited and provide the rationale for a switch to a genomic- and phenotypic-driven personalized medicine.

## Supplementary information

Supplementary information

Table S1

Table S2

Table S3

Table S4

Table S11

Table S12

Table S13

Table S14

Table S15

Table S16
