# Supplementary material for: Integrated genomic-metabolic classification of acute myeloid leukemia defines a subgroup with NPM1 and cohesin/DNA damage mutations
Source: Leukemia. 2021 Jun 30;35(10):2813–26. doi: 10.1038/s41375-021-01318-x (PMC8478658; doi:10.1038/s41375-021-01318-x)
Supplement: Supplementary file 1 — Supplementary information [file 41375_2021_1318_MOESM1_ESM.docx]

**Supplementary Information**

**Supplementary Methods**

**Sample preparation**

Buccal swab samples were collected with the Oragene Discover kit (DNA Genotek, Ottawa, Canada). Genomic DNA was extracted from leukemic, remission and buccal swab samples (AllPrep DNA/RNA/Protein Mini Kit and QIAcube, Qiagen, [Hilden, Germany](https://www.google.com/search?rlz=1C1GCEB_enIT823IT823&q=Hilden&stick=H4sIAAAAAAAAAOPgE-LUz9U3sDQ2z7JQAjON401yk7S0spOt9POL0hPzMqsSSzLz81A4VhmpiSmFpYlFJalFxYtY2Twyc1JS83awMgIAWRJGP1AAAAA&sa=X&ved=2ahUKEwi5-Ij0vJ_sAhXElqQKHao1CHMQmxMoATAYegQIDxAD), and Maxwell® 16 LEV DNA Blood Purification Kit and Maxwell MDx Instrument, Promega, [Madison, WI, USA](https://www.google.com/search?rlz=1C1GCEB_enIT823IT823&q=Madison+(Wisconsin)&stick=H4sIAAAAAAAAAOPgE-LUz9U3MKswKilR4gAx08qNKrW0spOt9POL0hPzMqsSSzLz81A4VhmpiSmFpYlFJalFxYtYhX0TUzKL8_MUNMIzi5Pz84oz8zR3sDICAJLnNeJcAAAA&sa=X&ved=2ahUKEwj_qPSAvZ_sAhUSwKQKHUY8DjIQmxMoATAZegQIDRAD), respectively), according to the manufacturer’s recommendations. Serum and urine samples were processed by centrifugation 30 min-to-2h after collection. Serum was prepared by centrifugation at 1500 x g at room temperature. Urine samples were centrifuged at 2500 x g at 4°C and filtered through a 0.22 μm syringe filter. Aliquots were stored at -80°C for nuclear magnetic resonance (NMR) analysis. AML sample immunophenotype was evaluated by flow cytometry using CD45, CD34, CD33 and CD14 antibodies (BD Biosciences, San Jose, CA, USA and FACSCanto II or Accuri C6, BD Biosciences). Leukocytes were enriched by separation on Ficoll density gradient. CD34^+^ and CD33^+^CD14^−^ cells from patient bone marrow mononuclear cells and CD34^+^ and CD33^+^ cells from cord blood and peripheral blood of healthy donors were freshly isolated (microbeads and AutoMacs Pro, Miltenyi Biotec, Bergisch Gladbach, Germany). Dried cell pellets (1x10^6^ cells) were frozen for metabolomic analysis.

**Nuclear magnetic resonance (NMR) spectra acquisition and analysis**

A 500 μL aliquot of serum sample was placed in a clean microfuge tube containing 130 μL of D2O-based phosphate buffer pH 7.4, 70 mM sodium azide (NaN3), 20 mM 4,4-dimethyl-4-silapentane-1-sulfonic acid (DSS-d6) as chemical shift reference standard and 20 mM 2-chloro pyrimidine-5-carboxylic acid (2CLPYR5CA) as reference standard. The mixture was homogenized by vortexing and 590 μL were transferred into 5x 178 mm (7”) 5 mm outer diameter NMR tubes (for Bruker Match holder). ^1^H-NMR spectra were recorded from serum and urine samples at 298 K with an AVANCE spectrometer (Bruker BioSpin, Fällanden, Switzerland) operating at a proton frequency of 600.13 MHz, equipped with an autosampler with 60 holders. ^1^H-NMR spectra were acquired by applying a standard spin echo Carr-Purcell-Meiboom-Gill (CPMG; cpmgpr1d.comp; Bruker BioSpin, Fällanden, Switzerland) pulse sequence with 256 scans (NS), 32768 data points (TD), a spectral width (SW) of 11.9705 ppm, an acquisition time (AQ) of 2.28 s, and a saturation time of 0.3 milliseconds (D20). A relaxation delay (D1) of 4 s was needed to reduce the signals arising from macromolecules. The data were Fourier transformed and phase and baseline corrections were automatically applied (TopSpin 3.0, Bruker BioSpin). Signals were assigned by comparing their chemical shift and multiplicity with Chenomx software data bank 8.1. 324 serum and 378 urine spectra passed quality control procedures (145 and 139 from controls, 179 and 139 from AML, respectively). Uniform binning was applied, resulting in 421 spectral features for each subject. Median Control Specter was used as reference for probabilistic quotient normalization and processing. During the quality control procedures, duplicate spectra (urine or serum samples from two different collection days) were compared, by taking into account diet, drugs, physical exercise data. Spectra showing unmatched peaks resulting from potential confounding factors (*e.g.* drugs) were excluded from downstream analysis.

**Learning methods for NMR data analysis**

Three different machine learning algorithms/classifiers were used for each task to perform cross-validated predictive modelling using latent components as inputs: Linear Kernel SVM, Random Forest Classifier, Ada Boosting Tree Classifier, with AdaBoost being the best performer across most of the tasks. Pipelines and algorithm scripts were implemented using Python 3.6 and SciKit.Learn module for machine learning routines. Feature-related scores from SVM classifier were used to extract the best subsets of latent components for plotting. The assumption is that the combination of the top 3 features contributing to SVM classification yields the best possible 3D space where group linear separability emerges. A similar approach is used when selecting the best latent components to be investigated for a given task.

**Ultrahigh Performance Liquid Chromatography-Tandem MS (UPLC-MS/MS)**

Proteins were precipitated with methanol under vigorous shaking for 2 min followed by centrifugation. Samples were placed briefly on a TurboVap® (Zymark Corporation, Hopkinton, MA, USA) to remove the organic solvent. The sample extract was dried and then reconstituted in solvents compatible with each of the four methods: i) for more hydrophilic compounds, the extract was gradient eluted from a C18 column (Waters UPLC BEH C18-2.1x100 mm, 1.7 µm) using water and methanol, containing 0.05% perfluoropentanoic acid (PFPA) and 0.1% formic acid (FA); ii) for more hydrophobic compounds, the extract was gradient eluted from the same aforementioned C18 column using methanol, acetonitrile, water, 0.05% PFPA and 0.01% FA; iii) basic extracts were obtained using basic negative ion optimized conditions and a separate dedicated C18 column, and then gradient eluted with methanol and water but with 6.5 mM Ammonium Bicarbonate at pH 8; iv) the fourth aliquot was analyzed via negative ionization following elution from a HILIC column (Waters UPLC BEH Amide 2.1x150 mm, 1.7 µm) using a gradient consisting of water and acetonitrile with 10 mM Ammonium Formate, pH 10.8. The MS analysis alternated between MS and data-dependent MSn scans using dynamic exclusion. The scan range varied slightly between methods but covered 70-1000 m/z (Metabolon, Morrisville, NC, USA).

**Lipidomics platform**

The TrueMass Lipomic and TrueMass Ceramides Panels were used for intracellular lipid detection (Metabolon). TrueMass Lipomic Panel: lipids were extracted using chloroform:methanol (2:1 v/v) in the presence of authentic internal standards. A solvent system consisting of petroleum ether/diethyl ether/acetic acid (80:20:1) was employed for the separation of neutral lipid classes. Individual phospholipid classes within each extract were separated using the Agilent Technologies 1100 Series LC (Agilent Technologies, Santa Clara, CA, USA). Each lipid class was transesterified in 1% sulfuric acid in methanol in a sealed vial under a nitrogen atmosphere at 100°C for 45 minutes. The resulting fatty acid methyl esters were extracted from the mixture with hexane containing 0.05% butylated hydroxytoluene and prepared for GC by sealing the hexane extracts under nitrogen. Fatty acid methyl esters were separated and quantified by capillary GC (Agilent Technologies 6890 Series GC) equipped with a 30 m DB 88 capillary column (Agilent Technologies) and a flame ionization detector.

**TrueMass Ceramides Panel**

Deuterium-labeled internal standards were added to the samples and the mixture was solubilized in methanol followed by a crash extraction. A bilayer was formed with the addition of KCl in water, and the organic layer was removed and concentrated under nitrogen. The extract was spun, filtered, and split into 2 injections (for ceramides and for sphingosines). The extract was injected onto an Agilent C8 column connected to an Agilent 1290 Infinity LC and ABI 4000 QTRAP. The analytes were ionized via positive electrospray and the mass spectrometer was operated in the tandem MS mode. The absolute concentration of each sphingolipid was determined by comparing the peak to that of the relevant internal standard.

**Somatic variant detection**

Average whole exome sequencing (WES) coverage of tumor and germline samples was 83.7X and 72.1X, respectively. Sequencing reads were quality control checked using FastQC ^1^ tool before and after trimming. AdapterRemoval (v1.5.4) ^2^ was employed to remove adapter sequences and adjust quality values, with the following parameters: --stats --trimns --trimqualities --minquality 20 --minlength 57. The sequence reads were then mapped to the human genome reference (human_g1k_v37) using BWA-MEM ^3^ (v0.7.12), with –M argument. Output SAM were converted to BAM with SAMtools ^4^ (v1.5) and sorted by coordinate with Picard SortSam (v1.119). Duplicates were marked and BAM indexed with Picard MarkDuplicates and BuildBamIndex, respectively. GATK ^5^ (v3.4-0) was used for local realignment around insertions/deletions (indels) and base quality scores recalibration, to improve the accuracy of the variant calling (RealignerTargetCreator and BaseRecalibrator with argument -ip 50. Resulting BAM files were analyzed by MuTect ^6^ (v1.1.4) and VarScan2 ^7^ (v2.3.9) algorithms for somatic variant calling; the former calls only single nucleotide variants (SNV), while the latter also calls small insertions/deletions (indels). Results of both tools were merged. Variants labelled as COVERED and KEEP by MuTect were kept. For VarScan2, BAM were pre-processed with SAMtools mpileup (with -B -q 1 arguments), then the somatic command of VarScan2 was run on the mpileup files, with the following parameters: --min-avg-qual 15 --strand_filter 1 --min-var-freq 0.05 --somatic-p-value 0.05 --tumor-purity 1 --normal-purity 0.8. In case of known tumor and normal sample purity, the arguments --tumor-purity and --normal-purity were adjusted accordingly. Both SNV and indels called by VarScan2 were processed with its somaticFilter command with the arguments: --min-coverage 1 --min-reads2 2 --min-var-freq 0.1. Resulting variants were annotated with ANNOVAR ^8^ tool using build version hg19.

**Downstream variant filtering**

Nuclear and mt variants were merged and filtered as follows:

1. only exonic variants were kept;
2. synonymous SNVs were removed;
3. variants of genes belonging to our blacklist (Table S1) ^9–11^ were removed;
4. variants falling inside regions of segmental duplication (SEGDUP) were removed, except for genes known to be cancer-related (Table S2);
5. variants called by VarScan2 and labelled as ‘Somatic’ were kept;
6. variants called by VarScan2 that passed somaticFilter were kept;
7. variants called by VarScan2 with strand bias (defined as variants having variant-supporting reads coming exclusively from one strand) in the tumor sample were removed;
8. Polymorphisms were defined as having minor allele frequency (MAF)≥0.01 according to Exome Sequencing Project (ESP, http://evs.gs.washington.edu/EVS) or Exome Aggregation Consortium (ExAC, http://exac.broadinstitute.org) or 1000 Genomes Project (http://www.internationalgenome.org) or dbSNP138 (www.ncbi.nlm.nih.gov/projects/SNP). Among total SNPs in dbSNP138, only around 81.4% were considered as polymorphisms, following these criteria: (i) the SNP has only one known alternative allele; (ii) the lower limit of the 99%CI of the MAF is ≥0.01 (to exclude SNPs derived from studies with small sample size, http://hgdownload.soe.ucsc.edu/goldenPath/hg19/database/snp138Common.txt.gz).

**Manual curation of variants**

Due to potential buccal swab contamination by tumor cells, some somatic variants were classified as ‘Germline’ by VarScan2 or, even if classified as ‘Somatic’, they did not pass the ‘Somatic filter’. We therefore rescued them by manual curation according to the following criteria:

- - - 1. passed previous filters up to step 4;

1. its normal VAF was between 0.05 and 0.25 and it belonged to a list of known myeloid-related genes ^12^;
2. the ratio between normal VAF and tumor VAF was ≤0.75 (or the difference between tumor VAF and normal VAF was ≥ 0.05);
3. its tumor VAF was > 0.05.

The final list of variants is reported in Table S12.

**Targeted next generation sequencing and DNA Sanger sequencing**

The tumor mutational landscape of patients who previously underwent allogeneic transplantation or did not undergo buccal swab collection (but donated peripheral blood or bone marrow sample), was analyzed by the targeted sequencing of a panel of myeloid genes (n=21, TruSight Myeloid Sequencing Panel by Illumina, San Diego, CA, USA or Myeloid SolutionTM by SOPHiA GENETICS, Losanna, Switzerland, and Illumina MiSeq). Reads from the TruSight Myeloid Sequencing Panel were aligned to the human reference genome (hg19) with BWA MEM (v0.7.12). GATK (v3.6-0) was used to recalibrate base qualities and realign mapped reads around indels, to ensure good call quality and to reduce the number of false positives. Somatic SNVs and small indels were called with VarScan2 (v2.3.9) algorithm with --min-coverage 10 --min-var-freq 0.01 parameters. Resulting variants were annotated with ANNOVAR. Coverage statistics was performed by DepthOfCoverage utility of GATK. BASH and R custom scripts were used to obtain the list of low coverage (<50X) regions per sample. FASTQ sequencing files of SOPHiA Myeloid SolutionTM panel were uploaded onto the SOPHiA DDM® platform (v.4). Hg19 was used as the reference for sequence alignment. Capillary electrophoresis was performed to validate *NPM1* mutations ^13^ and detect *FLT3*-ITD ^14^.

**Analysis of data from The Cancer Genome Atlas (TCGA) and BEAT AML cohorts**

WES, RNA-seq and clinical data of the TCGA-LAML (n=157 WES; n= 66 RNA-seq from *NPM1*-mut AML) and BEAT AML cohorts (n=91 WES, n= 121 RNA-seq, including *NPM1*-mut AML initial diagnosis and relapse), were obtained from the GDC Data Portal (https://gdc.cancer.gov) and from Tyner *et al*. ^12^, respectively. Drug sensitivity data were retrieved from the BEAT AML study.

**RNA-seq data analysis**

Differential Gene Expression (DGE) analysis on RNA-seq data was performed with edgeR (v.3.24.1, R3.5.1) on log2 Counts Per Million (CPM) adjusted with weighted Trimmed Mean of M-values (TMM) method (hereinafter referred to as CPM). Given that available data for the BEAT AML dataset included normalized expression data (CPM and RPKM, with no read counts), read counts from the TCGA dataset were transformed to CPM (TMM) using calcNormFactors(method=”TMM”) function in edgeR, to keep them homogeneous. Genes with CPM≥1 across every sample were filtered out before log2 transformation (0.25 prior was added to CPM values). As PCA revealed a dataset-related separation (Fig.S5), the two cohorts were separately analyzed in order to define a core of common Differentially Expressed Genes (DEGs, |fold change|≥2 and *p*≤0.05). DEGs from both datasets (*p*≤0.05) were analyzed with GSEA Preranked (through gseapy v0.9.16, https://pypi.org/project/gseapy/) with default settings (aside from min_size=1), using log2 fold change (FC) to rank the genes. Heatmaps were generated using ComplexHeatmap (v.2.0.0) and circlize packages (v.0.4.8) on R v.3.6.1.

**Metabolic model**

Six different metabolic models were used throughout this work. Recon3D was chosen as the base model and reference for all the others. It was also the basis for mapping the metabolite from MS analysis and the gene symbols (corresponding to the Entrez IDs) used to label genes in constraint based modelling. All these changes to the original models were preserved in scripts, along with the databases used for Entrez IDs and MS labels. To identify the best fitting metabolic model to our experimental setting, we evaluated hematological and myeloid-related cell-specific metabolic reconstructions derived from MinMax and mCADRE algorithms. As the cell-specific models came from different sources and had base models other than Recon 3D, the models were modified to reconcile metabolite and reaction names. Optimization problems were run with the Cobra Toolbox using cplex as the LP solver and also the MatLab Optimization Toolbox.

**Constraint-based metabolic network analysis**

We selected a hematopoietic model derived from Recon2 via the integration of proteomic data for bone marrow hematopoietic cells from the Human Protein Atlas. A genetic perturbation was characterized by a gene and a direction of perturbation (up/down). All reactions involving any downregulated genes in their regulatory rules were set to zero flux, while for those involving upregulated genes, the minimum flux was set to a non-zero value, which we will informally call "cut depth". This type of specification can lead to situations where no mass balanced flux distribution can satisfy them. In this implementation, when a new perturbation set is specified, its maximal perturbation sets (which have a non-zero maximum cut depth value) are found, along with the minimal sets of perturbations, which are incompatible. The effect, if any, on the metabolic network is then calculated by maximizing or minimizing the reaction rates (flux variability analysis, FVA) for the reactions. For the metabolites, the corresponding mass balance is relaxed and the corresponding row of the stoichiometric matrix is used as the objective vector. This is analogous to adding a sink or source reaction and maximizing their flux.

**Metabolic network reconstruction**

The Reaction-Reaction network was generated using the Metabolite-Reactions (1581x2274) stoichiometric matrix mapped in the bone marrow-Recon model. Two reactions are linked if a metabolite produced in one is consumed in the other, resulting in a directed network (source:production, target:consumption). Moreover, if a perturbed metabolite is produced in a reaction and consumed in the other, those reactions are marked as perturbed. In this way, a subgraph of altered reaction can easily be extracted and analyzed. Network building and analyses were performed using Python 3.6 NetworkX package, while visualization and graphic processing were obtained using the Cytoscape 3.7.2 framework. Genes involved in the identified reactions were retrieved from Recon3D and their interconnection was evaluated by protein-protein interaction analysis on STRING (https://string-db.org).

**Supplementary References**

1 Andrews S. *FastQC - A quality control tool for high throughput sequence data. http://www.bioinformatics.babraham.ac.uk/projects/fastqc/*. 2010 doi:citeulike-article-id:11583827.

2 Lindgreen S. AdapterRemoval: Easy cleaning of next-generation sequencing reads. *BMC Res Notes* 2012. doi:10.1186/1756-0500-5-337.

3 Li H, Durbin R. Fast and accurate short read alignment with Burrows-Wheeler transform. *Bioinformatics* 2009. doi:10.1093/bioinformatics/btp324.

4 Li H, Handsaker B, Wysoker A, Fennell T, Ruan J, Homer N *et al.* The Sequence Alignment/Map format and SAMtools. *Bioinformatics* 2009. doi:10.1093/bioinformatics/btp352.

5 Depristo MA, Banks E, Poplin R, Garimella K V., Maguire JR, Hartl C *et al.* A framework for variation discovery and genotyping using next-generation DNA sequencing data. *Nat Genet* 2011. doi:10.1038/ng.806.

6 Cibulskis K, Lawrence MS, Carter SL, Sivachenko A, Jaffe D, Sougnez C *et al.* Sensitive detection of somatic point mutations in impure and heterogeneous cancer samples. *Nat Biotechnol* 2013. doi:10.1038/nbt.2514.

7 Koboldt DC, Larson DE, Wilson RK. Using varscan 2 for germline variant calling and somatic mutation detection. *Curr Protoc Bioinforma* 2013. doi:10.1002/0471250953.bi1504s44.

8 Wang K, Li M, Hakonarson H. ANNOVAR: Functional annotation of genetic variants from high-throughput sequencing data. *Nucleic Acids Res* 2010. doi:10.1093/nar/gkq603.

9 Fuentes Fajardo K V., Adams D, Mason CE, Sincan M, Tifft C, Toro C *et al.* Detecting false-positive signals in exome sequencing. *Hum Mutat* 2012. doi:10.1002/humu.22033.

10 Kwak SH, Chae J, Choi S, Kim MJ, Choi M, Chae JH *et al.* Findings of a 1303 Korean whole-exome sequencing study. *Exp Mol Med* 2017. doi:10.1038/emm.2017.142.

11 Lawrence MS, Stojanov P, Mermel CH, Robinson JT, Garraway LA, Golub TR *et al.* Discovery and saturation analysis of cancer genes across 21 tumour types. *Nature* 2014. doi:10.1038/nature12912.

12 Tyner JW, Tognon CE, Bottomly D, Wilmot B, Kurtz SE, Savage SL *et al.* Functional genomic landscape of acute myeloid leukaemia. *Nature* 2018. doi:10.1038/s41586-018-0623-z.

13 Lin LI, Lin TC, Chou WC, Tang JL, Lin DT, Tien HF. A novel fluorescence-based multiplex PCR assay for rapid simultaneous detection of CEBPA mutations and NPM mutations in patients with acute myeloid leukemias [8]. Leukemia. 2006. doi:10.1038/sj.leu.2404331.

14 Thiede C, Steudel C, Mohr B, Schaich M, Schäkel U, Platzbecker U *et al.* Analysis of FLT3-activating mutations in 979 patients with acute myelogenous leukemia: Association with FAB subtypes and identification of subgroups with poor prognosis. *Blood* 2002. doi:10.1182/blood.V99.12.4326.

15 MacIntyre DA, Jiménez B, Lewintre EJ, Martín CR, Schäfer H, Ballesteros CG *et al.* Serum metabolome analysis by 1H-NMR reveals differences between chronic lymphocytic leukaemia molecular subgroups. *Leukemia* 2010. doi:10.1038/leu.2009.295.

16 Wojtowicz W, Chachaj A, Olczak A, Ząbek A, Piątkowska E, Rybka J *et al.* Serum NMR metabolomics to differentiate haematologic malignancies. *Oncotarget* 2018. doi:10.18632/oncotarget.25311.

17 Giskeødegård GF, Madssen TS, Euceda LR, Tessem MB, Moestue SA, Bathen TF. NMR-based metabolomics of biofluids in cancer. NMR Biomed. 2019. doi:10.1002/nbm.3927.

18 Hasim A, Ma H, Mamtimin B, Abudula A, Niyaz M, Zhang LW *et al.* Revealing the metabonomic variation of EC using 1 H-NMR spectroscopy and its association with the clinicopathological characteristics. *Mol Biol Rep* 2012. doi:10.1007/s11033-012-1764-z.

19 Emadi A, Jun SA, Tsukamoto T, Fathi AT, Minden MD, Dang C V. Inhibition of glutaminase selectively suppresses the growth of primary acute myeloid leukemia cells with IDH mutations. *Exp Hematol* 2014. doi:10.1016/j.exphem.2013.12.001.

**Supplementary Tables**

**Table S1.** **Blacklisted genes.** Summary of all genes to be removed from the final variant list based on information from previous studies.

**Table S2. Rescued genes.** Summary of all genes to be reintroduced in the final variant list based on variant features and information from previous studies, as outlined in the supplementary methods.

**Table S3. Serum leukemia assignments of major resonances detected in ^1^H-NMR spectra from human serum samples**. Chemical shifts (δ) used for the identification of compounds are reported together with the chemical group assignment and peak multiplicity. All the chemical shifts were referenced to the H1 of alfa-glucose at 5.26 ppm ^15,16^ (s-singlet; d-doublet; dd-double doublet, t-triplet; q-quartet; m-multiplet; LDL-low density lipid; VLDL- very low density lipid; NAC-N-acetyl signals from α1-acid glycoproteins).

**Table S4. Urine leukemia assignments of major resonances detected in ^1^H-NMR spectra from human urine samples**. Chemical shifts (δ) used for the identification of compounds are reported together with the chemical group assignment and peak multiplicity. All the chemical shifts were referenced to the DSS at 0.00 ppm ^17,18^ (s-singlet; d-doublet; dd-double doublet, t-triplet; q-quartet; m-multiplet).

**Table S5. Spearman correlation coefficient of age or gender and principal components in urine and serum NMR.**

|  | **Age** | | **Gender** | |
| --- | --- | --- | --- | --- |
|  | **Serum** | **Urine** | **Serum** | **Urine** |
| **PC1** | 0.036107 | 0.079113 | -0.03625 | -0.09505 |
| **PC2** | -0.01198 | 0.163624 | -0.06494 | -0.24853 |
| **PC3** | 0.257916 | -0.04961 | 0.031996 | 0.061732 |
| **PC4** | 0.308376 | 0.36186 | -0.11975 | -0.02851 |
| **PC5** | -0.17938 | 0.027464 | 0.030511 | -0.10209 |
| **PC6** | 0.113539 | 0.014142 | -0.17233 | -0.12378 |
| **PC7** | -0.02777 | -0.09573 | 0.057309 | -0.18044 |
| **PC8** | -0.04371 | -0.06614 | 0.211955 | 0.099348 |
| **PC9** | 0.029367 | 0.301943 | 0.128996 | -0.0891 |
| **PC10** | 0.007273 | -0.11202 | -0.07121 | -0.03336 |

**Table S6. Spearman correlation coefficient of age/gender and metabolites altered in serum/urine of AML patients.**

| **Metabolite** | **Age** | | **Gender** | |
| --- | --- | --- | --- | --- |
|  | **Serum** | **Urine** | **Serum** | **Urine** |
| 3-Hydroxybutyrate | 0.102135 |  | -0.08809 |  |
| Glycerol of Lipids | 0.232803 |  | 0.038881 |  |
| Glucose | 0.162726 |  | 0.066084 |  |
| Glutamine | -0.184395 |  | -0.0054 |  |
| Lactate | -0.007234 |  | 0.004995 |  |
| Low Density/Very Low Density Lipids1 | -0.138556 |  | -0.245504 |  |
| Low Density/Very Low Density Lipids2 | -0.00236 |  | -0.031591 |  |
| N-acetylglycoproteins (1 & 2) | 0.018596 |  | -0.032468 |  |
| Poly-Unsaturated Fatty Acids | 0.02501 |  | -0.013703 |  |
| Pyruvate+Succinate | 0.056592 |  | -0.049411 |  |
| Threonine | -0.295995 |  | -0.020453 |  |
| Valine | 0.021784 |  | 0.092207 |  |
| PhenylAlanine | 0.08492 | 0.171833 | 0.069527 | -0.057614 |
| 3-Aminobutyrate |  | 0.109685 |  | -0.093399 |
| Citrate |  | -0.169339 |  | -0.121313 |
| Creatinine |  | -0.335887 |  | 0.400915^*^ |
| Glycine |  | -0.140462 |  | -0.116097 |
| Hippurate |  | 0.07538 |  | 0.005354 |
|  |  |  |  |  |
| ^*^ Kruskal-Wallis *p*= 0.178. |  |  |  |  |

**Table S7. Metabolic pathways significantly deregulated in CD34^+^ AML cells compared with CD34^+^ cord blood cells.**

| **Metabolic pathway** | ***p*-value** | **FDR*** | **Impact^$^** |
| --- | --- | --- | --- |
| Citrate cycle (TCA cycle) | 5.10E-08 | 1.56E-06 | 0.3693 |
| Glyoxylate and dicarboxylate metabolism | 4.19E-08 | 1.56E-06 | 0.06354 |
| Pentose phosphate pathway | 8.88E-07 | 1.81E-05 | 0.15737 |
| Glycine, serine and threonine metabolism | 6.51E-06 | 9.93E-05 | 0.45021 |
| Linoleic acid metabolism | 1.26E-05 | 0.0001535 | 0.65625 |
| D-Arginine and D-ornithine metabolism | 9.94E-05 | 0.00075818 | 0.5 |
| Ascorbate and aldarate metabolism | 0.00016084 | 0.0010902 | 0.17973 |
| Glycolysis or Gluconeogenesis | 0.00026085 | 0.0015912 | 0.1988 |
| Propanoate metabolism | 0.0003562 | 0.0019753 | 0.08634 |
| Primary bile acid biosynthesis | 0.00040359 | 0.0020516 | 0.07195 |
| Tyrosine metabolism | 0.00049 | 0.0022993 | 0.04724 |
| Phenylalanine metabolism | 0.00069807 | 0.0030416 | 0.11906 |
| Cysteine and methionine metabolism | 0.0012047 | 0.0037946 | 0.45685 |
| Pyruvate metabolism | 0.0012507 | 0.0037946 | 0.3201 |
| Aminoacyl-tRNA biosynthesis | 0.0011212 | 0.0037946 | 0.22536 |
| Porphyrin and chlorophyll metabolism | 0.001113 | 0.0037946 | 0.03338 |
| Methane metabolism | 0.0013063 | 0.0037946 | 0.01751 |
| Lysine degradation | 0.0013998 | 0.0038813 | 0.21735 |
| Pantothenate and CoA biosynthesis | 0.0014882 | 0.0039469 | 0.253 |
| Taurine and hypotaurine metabolism | 0.0016605 | 0.0041814 | 0.46583 |
| Purine metabolism | 0.0017699 | 0.0041814 | 0.34865 |
| beta-Alanine metabolism | 0.0017822 | 0.0041814 | 0.32319 |
| Nicotinate and nicotinamide metabolism | 0.0019964 | 0.0045105 | 0.0794 |
| Nicotinate and nicotinamide metabolism | 0.0021696 | 0.0047267 | 0.29398 |
| Glutathione metabolism | 0.0024201 | 0.0050906 | 0.03307 |
| Arginine and proline metabolism | 0.0029522 | 0.0059733 | 0.45158 |
| Valine, leucine and isoleucine biosynthesis | 0.0030356 | 0.0059733 | 0.06148 |
| Glycerophospholipid metabolism | 0.0034377 | 0.0062356 | 0.50791 |
| Butanoate metabolism | 0.0034756 | 0.0062356 | 0.12061 |
| Glycerolipid metabolism | 0.0044288 | 0.0077188 | 0.02774 |
| Fructose and mannose metabolism | 0.0051377 | 0.0084702 | 0.05002 |
| Nitrogen metabolism | 0.0050411 | 0.0084702 | 0.0083 |
| Starch and sucrose metabolism | 0.0062131 | 0.0097179 | 0.25776 |
| Pyrimidine metabolism | 0.0084345 | 0.012863 | 0.33052 |
| Lysine biosynthesis | 0.0088941 | 0.013233 | 0.09993 |
| Alanine, aspartate and glutamate metabolism | 0.013122 | 0.017452 | 0.75404 |
| Amino sugar and nucleotide sugar metabolism | 0.012572 | 0.017452 | 0.22432 |
| Galactose metabolism | 0.01316 | 0.017452 | 0.08912 |
| Steroid hormone biosynthesis | 0.02164 | 0.028086 | 0.00391 |
| Sphingolipid metabolism | 0.02214 | 0.028137 | 0.54746 |
| Pentose and glucuronate interconversions | 0.029154 | 0.036293 | 0.18622 |
|  |  |  |  |
| * False discovery rate (FDR)≤0.05 |  |  |  |
| ^$^ Impact>0 |  |  |  |

**Table S8. Metabolic pathways deregulated in CD33^+^ AML cells compared with CD33^+^ peripheral blood cells from controls.**

| **Metabolic pathway** | ***p*-value^*^** | **FDR^*^** | **Impact^*^** |
| --- | --- | --- | --- |
| Galactose metabolism | 0.0037609 | 0.16627 | 0.08912 |
| Taurine and hypotaurine metabolism | 0.012755 | 0.16627 | 0.46583 |
| Pentose phosphate pathway | 0.013833 | 0.16627 | 0.15737 |
| Cysteine and methionine metabolism | 0.014517 | 0.16627 | 0.45685 |
| Glutathione metabolism | 0.014746 | 0.16627 | 0.29398 |
| Glycerolipid metabolism | 0.018786 | 0.16627 | 0.02774 |
| Ascorbate and aldarate metabolism | 0.01908 | 0.16627 | 0.17973 |
| Primary bile acid biosynthesis | 0.026449 | 0.18415 | 0.07195 |
| Steroid hormone biosynthesis | 0.029412 | 0.18415 | 0.00391 |
| Sphingolipid metabolism | 0.037789 | 0.18415 | 0.54746 |
| Purine metabolism | 0.038785 | 0.18415 | 0.34865 |
| Alanine, aspartate and glutamate metabolism | 0.03959 | 0.18415 | 0.75404 |
| Tryptophan metabolism | 0.04079 | 0.18415 | 0.16587 |
| Propanoate metabolism | 0.042263 | 0.18415 | 0.08634 |
|  |  |  |  |
| * *p*-value ≤0.05, false discovery rate (FDR)≤0.2, impact>0 (these cut offs were chosen due to the low number of CD33^+^ AML cases) | | | |

**Table S9. Metabolic pathways deregulated in CD34^+^/CD33^+^ AML cells compared with CD34^+^ cord blood/CD33^+^ peripheral blood cells.**

| **Metabolic pathway** | ***p-*value** | **FDR^*^** | **Impact^*^** |
| --- | --- | --- | --- |
| Pentose phosphate pathway^$,&^ | 4.50E-07 | 1.37E-05 | 0.15737 |
| Glyoxylate and dicarboxylate metabolism^$^ | 3.48E-07 | 1.37E-05 | 0.06354 |
| Glycine, serine and threonine metabolism^$^ | 8.64E-06 | 0.00013176 | 0.45021 |
| Citrate cycle (TCA cycle)^$^ | 8.12E-06 | 0.00013176 | 0.3693 |
| D-Arginine and D-ornithine metabolism^$^ | 0.00014371 | 0.0014611 | 0.5 |
| Ascorbate and aldarate metabolism^$,&^ | 0.0004452 | 0.0038796 | 0.08139 |
| Primary bile acid biosynthesis^$,&^ | 0.00054541 | 0.0041587 | 0.07195 |
| Linoleic acid metabolism^$^ | 0.00072128 | 0.0048887 | 0.65625 |
| Sulfur metabolism | 0.0013252 | 0.0080839 | 0.03307 |
| Purine metabolism^$,&^ | 0.0014636 | 0.0081161 | 0.34865 |
| Taurine and hypotaurine metabolism^$,&^ | 0.0021511 | 0.0089126 | 0.46583 |
| Steroid hormone biosynthesis^$,&^ | 0.0020282 | 0.0089126 | 0.00391 |
| Methane metabolism^$^ | 0.0026407 | 0.010068 | 0.01751 |
| Cysteine and methionine metabolism^$,&^ | 0.0033691 | 0.012089 | 0.4495 |
| Nicotinate and nicotinamide metabolism^$^ | 0.0049733 | 0.015168 | 0.0794 |
| Lysine degradation^$^ | 0.0053555 | 0.015556 | 0.21735 |
| Valine, leucine and isoleucine biosynthesis^$^ | 0.005865 | 0.016262 | 0.06148 |
| Sphingolipid metabolism^$,&^ | 0.0070319 | 0.016544 | 0.54746 |
| Pyrimidine metabolism^$^ | 0.0068651 | 0.016544 | 0.33052 |
| Aminoacyl-tRNA biosynthesis^$^ | 0.0066344 | 0.016544 | 0.22536 |
| Nitrogen metabolism^$^ | 0.0070517 | 0.016544 | 0.0083 |
| Glycerolipid metabolism^$,&^ | 0.0078101 | 0.017015 | 0.02774 |
| Tryptophan metabolism^$,&^ | 0.0084682 | 0.017812 | 0.16587 |
| Galactose metabolism^$,&^ | 0.0099832 | 0.020299 | 0.08912 |
| Pantothenate and CoA biosynthesis^$^ | 0.012233 | 0.02332 | 0.253 |
| Valine, leucine and isoleucine degradation | 0.012026 | 0.02332 | 0.02232 |
| Glycerophospholipid metabolism^$^ | 0.013016 | 0.023683 | 0.50791 |
| Lysine biosynthesis^$^ | 0.0132 | 0.023683 | 0.09993 |
| Glutathione metabolism^$,&^ | 0.015419 | 0.026873 | 0.04893 |
| beta-Alanine metabolism^$^ | 0.016282 | 0.02759 | 0.32319 |
| Arginine and proline metabolism^$^ | 0.020567 | 0.033016 | 0.45158 |
| Fructose and mannose metabolism^$^ | 0.023008 | 0.035986 | 0.05002 |
| Arachidonic acid metabolism | 0.02444 | 0.036362 | 0.21669 |
| Pyruvate metabolism^$^ | 0.027125 | 0.039396 | 0.22982 |
| Ubiquinone and other terpenoid-quinone biosynthesis | 0.032881 | 0.046644 | 0.00069 |
| Glycolysis or Gluconeogenesis^$^ | 0.034656 | 0.048046 | 0.1988 |
|  |  |  |  |
| ^*^ False discovery rate (FDR)≤0.05, impact>0 |  |  |  |
| ^$^ found in the comparison between CD34^+^ AML and CD34^+^ cord blood cells | | |  |
| ^&^ found in the comparison between CD33^+^ AML and CD33^+^ peripheral blood cells | | |  |

**Table S10. Integrated data of metabolic alterations in the biofluid of AML patients and in the leukemic cells.**

| **Metabolite** | **Biofluid** | **CD34^+^ AML** | **CD33^+^ AML** | **CD34^+^/33^+^ AML** |
| --- | --- | --- | --- | --- |
| Glycerol of Lipids^$^ | ↑ serum | ↓ | ↑ GPG | ↓ glycerol 3P |
| Glucose | ↑ serum | - | - | ↓ |
| Glutamine | ↓ serum | ↑ | - | - |
| Lactate | ↑ serum | ↓ | ↓ | ↓ |
| Poly-UFA | ↑ serum | ↓ (some) | ↓ (some) | ↓ (some) |
| Pyruvate+Succinate | ↑ serum | ↓ succinate | - | - |
| Threonine | ↓ serum | ↓ | - | ↓ |
| Valine | ↑ serum | - | - | - |
| PhenylAlanine | ↑ serum/urine | - | - | ↓ |
| Citrate | ↓ urine | ↓ | ↓ | ↓ |
| Glycine | ↓ urine | ↓ | - | - |
|  |  |  |  |  |
| ^*^ All the data are reported as AML *versus* control; ↑: up; ↓: down; -: no significant difference | | | |  |
| ^$^ GPG: glycerophosphoglycerol; glycerol 3P: glycero 3-phosphate | | |  |  |

**Table S11**. **Metabolites showing a differential distribution across the three MS metabolic clusters.** (FC: fold change; FDR: false discovery rate; ns: not significant).

**Table S12. Variants from WES and targeted NGS.** List of variants obtained from variant calling, filtering and validation.

**Table S13. *In silico* prediction of metabolic alterations induced by *IDH* mutation in the model.** Columns indicate (left to right): the network object short and long name, the object type (metabolite), the type of function being tested (maximum production or consumption), the maximum value for the function in the perturbed model, and the baseline value for the function in the model without perturbations.

**Table S14. Model of metabolite and reactions perturbation of *NPM1*-mut AML.** Columns indicate (left to right): the network object short and long name, the object type (metabolite or reaction), the type of function being tested (maximum production or consumption for metabolites, maximum or minimum flux (reaction rate) for reactions), the maximum value for the function in the perturbed model, and the baseline value for the function in the model without perturbations. (TYMP upregulation could not be modelled, since its related reactions cannot carry flux in the original model).

**Table S15. Model of metabolite and reactions perturbation of *NPM1*/cohesin-mut AML.** Columns indicate (left to right): the network object short and long name, the object type (metabolite or reaction), the type of function being tested (maximum production or consumption for metabolites, maximum or minimum flux (reaction rate) for reactions), the maximum value for the function in the perturbed model, and the baseline value for the function in the model without perturbations. (TYMP upregulation could not be modelled, since its related reactions cannot carry flux in the original model and AOC1-related reaction could not be forced due to blocking of ALDH3A2-related ones).

**Table S16. *NPM1*/cohesin-mut-specific metabolite and reactions perturbations.** List of metabolites with disrupted maximal production or consumption and reactions with changed minimum or maximum flux in *NPM1*/cohesin-mut model that did not show the same perturbation type in the *NPM1*-mut model. Columns indicate (left to right): the network object short and long name, the object type (metabolite or reaction), the type of function being tested (maximum production or consumption for metabolites, maximum or minimum flux (reaction rate) for reactions), the maximum value for the function in the perturbed model, and the baseline value for the function in the model without perturbations.

**Table S17. Pathway enrichment analysis of genes involved in *NPM1*/cohesin-mut-specific metabolic network of reactions.**

| **KEGG ID** | **Pathway** | **FDR** | | **Genes** | |  |  |
| --- | --- | --- | --- | --- | --- | --- | --- |
| hsa00071 | Fatty acid degradation | 4.48E-18 | | *ACSL1, ACSL3, ACSL4, ACSL5, ACSL6, ALDH1B1, ALDH2, ALDH3A2, ALDH7A1, ALDH9A1, CPT1A, GCDH* | |  |  |
| hsa00410 | beta-Alanine metabolism | 9.42E-18 | | *ALDH1A3, ALDH1B1, ALDH2, ALDH3A1, ALDH3A2, ALDH3B1, ALDH3B2, ALDH7A1, ALDH9A1, HIBCH, UPB1* | |  |  |
| hsa00010 | Glycolysis / Gluconeogenesis | 2.29E-16 | | *ALDH1A3, ALDH1B1, ALDH2, ALDH3A1, ALDH3A2, ALDH3B1, ALDH3B2, ALDH7A1, ALDH9A1, ALDOB, LDHB, PKLR* | |  |  |
| hsa00620 | Pyruvate metabolism | 9.12E-10 | | *ALDH1B1, ALDH2, ALDH3A2, ALDH7A1, ALDH9A1, LDHB, PKLR* | |  |  |
| hsa00380 | Tryptophan metabolism | 9.50E-10 | | *ALDH1B1, ALDH2, ALDH3A2, ALDH7A1, ALDH9A1, GCDH, TPH1* | |  |  |
| hsa00280 | Valine, leucine and isoleucine degradation | 2.46E-09 | | *AACS, ALDH1B1, ALDH2, ALDH3A2, ALDH7A1, ALDH9A1, HIBCH* | |  |  |
| hsa01212 | Fatty acid metabolism | 2.46E-09 | | *ACSL1, ACSL3, ACSL4, ACSL5, ACSL6, CPT1A, PECR* | |  |  |
| hsa00053 | Ascorbate and aldarate metabolism | 4.34E-09 | | *ALDH1B1, ALDH2, ALDH3A2, ALDH7A1, ALDH9A1, UGT2B10* | |  |  |
| hsa00061 | Fatty acid biosynthesis | 8.32E-09 | | *ACSL1, ACSL3, ACSL4, ACSL5, ACSL6* | |  |  |
| hsa00330 | Arginine and proline metabolism | 7.36E-08 | | *ALDH1B1, ALDH2, ALDH3A2, ALDH7A1, ALDH9A1, NOS2* | |  |  |
| hsa00240 | Pyrimidine metabolism | 1.71E-07 | | *NME1, NME2, NME3, NME6, NME7, PNP, UPB1* | |  |  |
| hsa00310 | Lysine degradation | 1.95E-07 | | *ALDH1B1, ALDH2, ALDH3A2, ALDH7A1, ALDH9A1, GCDH* | |  |  |
| hsa00230 | Purine metabolism | 3.12E-07 | | *DGUOK, NME1, NME2, NME3, NME6, NME7, PKLR, PNP* | |  |  |
| hsa00360 | Phenylalanine metabolism | 1.70E-06 | | *ALDH1A3, ALDH3A1, ALDH3B1, ALDH3B2* | |  |  |
| hsa00561 | Glycerolipid metabolism | 5.36E-06 | | *ALDH1B1, ALDH2, ALDH3A2, ALDH7A1, ALDH9A1* | |  |  |
| hsa00350 | Tyrosine metabolism | 2.04E-05 | | *ALDH1A3, ALDH3A1, ALDH3B1, ALDH3B2* | |  |  |
| hsa00480 | Glutathione metabolism | 0.0017 | | *GGCT, GGT1, GGT6* | |  |  |
| hsa00430 | Taurine and hypotaurine metabolism | 0.0019 | | *GGT1, GGT6* | |  |  |
| hsa00190 | Oxidative phosphorylation | 0.0021 | | *NDUFA3, NDUFS4, NDUFS7, PPA2* | |  |  |
| hsa00790 | Folate biosynthesis | 0.0083 | | *DHFR, TPH1* | |  |  |
| hsa00760 | Nicotinate and nicotinamide metabolism | 0.0106 | | *CD38, PNP* | |  |  |
| hsa01200 | Carbon metabolism | 0.0134 | | *ALDOB, HIBCH, PKLR* | |  |  |
| hsa01230 | Biosynthesis of amino acids | 0.0441 | | *ALDOB, PKLR* | |  |  |
|  |  | |  | |  | | |
| FDR: false discovery rate | | |  | |  | |  |

**Supplementary Figures**

sPC1

sPC2

sPC3

sPC4

sPC5

sPC6

sPC7

sPC8

sPC9

sPC10

AML

CTRL

sPC1

sPC2

sPC3

sPC4

sPC5

sPC6

sPC7

sPC8

sPC9

sPC10

**Type of lesion**

**No. of lesions**

Missense

Truncating

Inframe

Splice

Translocation

**Fig.1. Relationship between each pair of serum PCs.** The diagonal plots are kernel density estimation of the marginal distribution of the scores of each single serum (s)PC (n=10). The other plots are scatter plots representing pairwise bivariate distributions (AML: acute myeloid leukemia; CTRL: control; PC: principal component, s: serum).

uPC1

uPC2

uPC3

uPC4

uPC5

uPC6

uPC7

uPC8

uPC9

uPC10

uPC1

uPC2

uPC3

uPC4

uPC5

uPC6

uPC7

uPC8

uPC9

uPC10

AML

CTRL

**Figure S2. Relationship between each pair of urine PCs.** The diagonal plots are kernel density estimation of the marginal distribution of the scores of each single urine PC (n=10). The other plots are scatter plots representing pairwise bivariate distributions (AML: acute myeloid leukemia; CTRL: control; PC: principal component, u: urine).

**
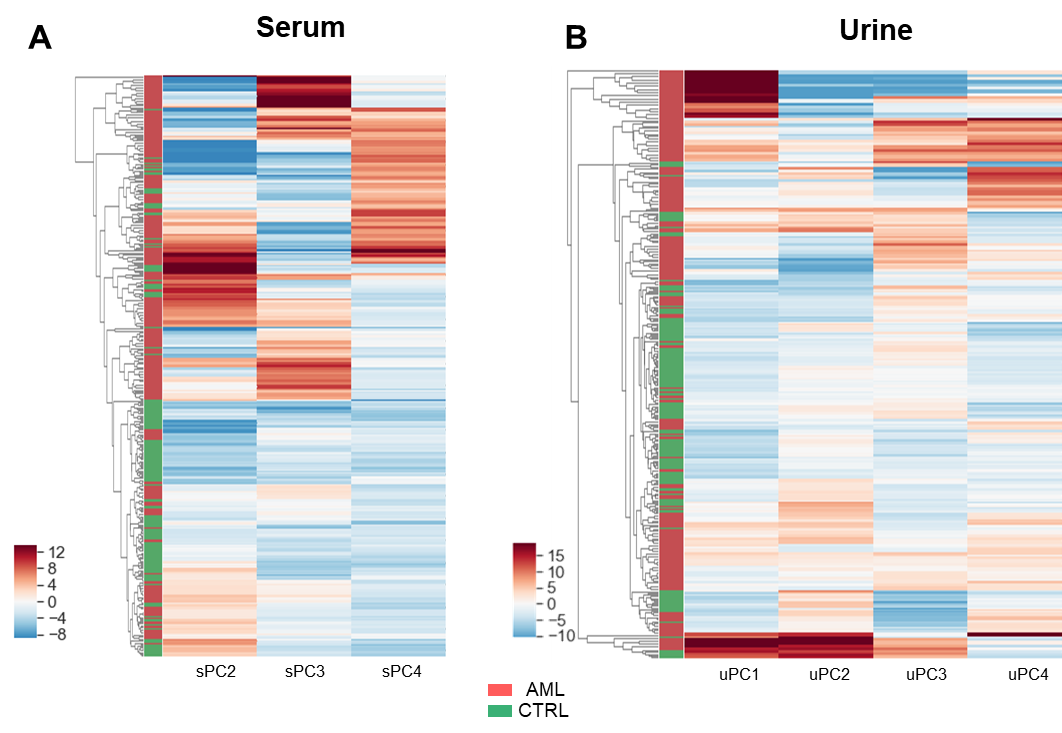
**

**Figure S3. Hierarchical clustering of AML and healthy controls on single biofluid NMR principal components.** Clustering on (**A**) serum components (sPC, n=3) and (**B**) urine components (uPC, n=4). This is the best combination of features leading to maximum accuracy and minimum number of features needed when the biofluid profiles are merged. Colors indicate the score on each PC.

**
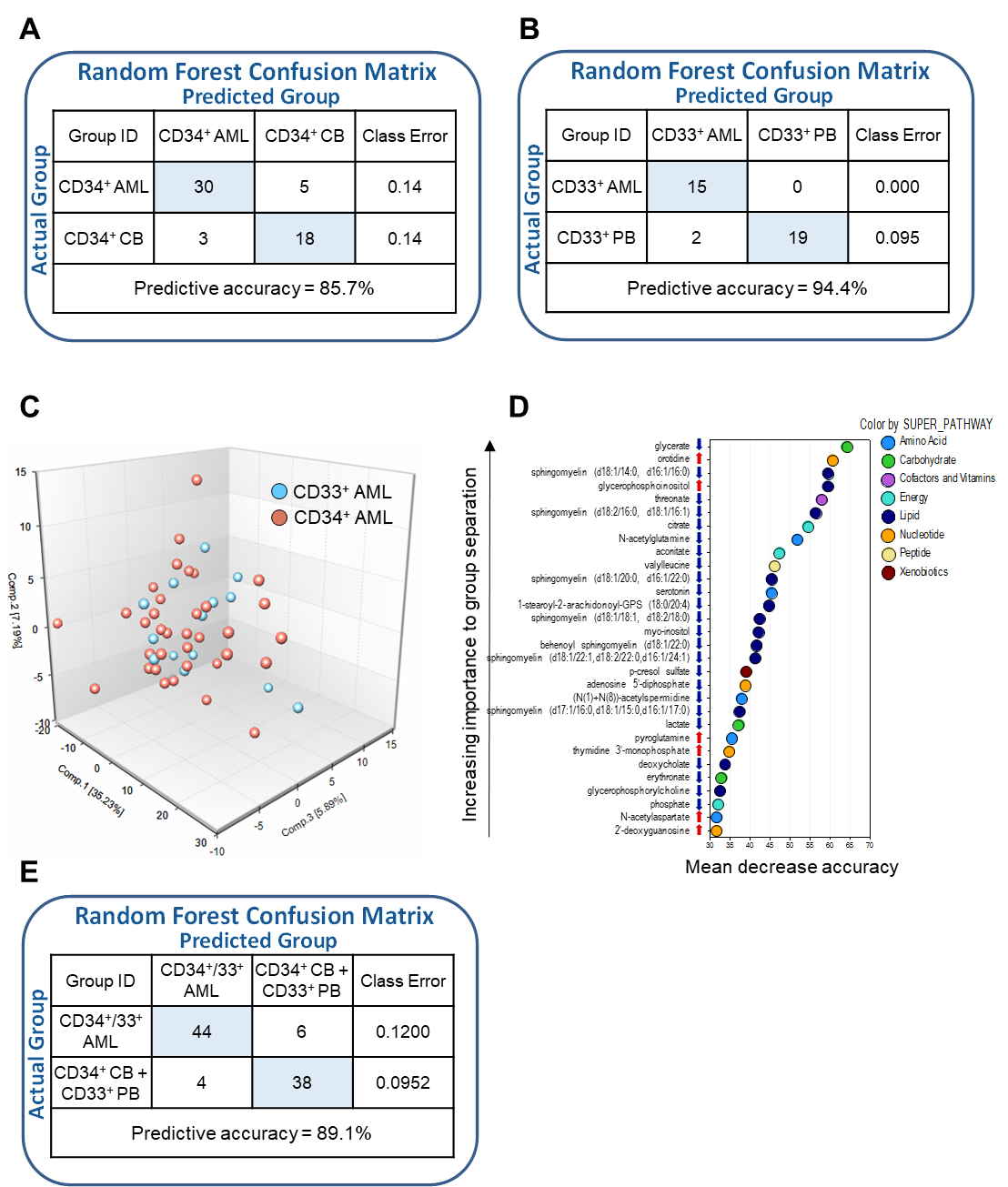
Figure S4. Separation between AML cell subpopulations and within AML and healthy control populations according to the intracellular metabolic profile.** (**A, B**) Random Forest classification comparing CD34^+^ AML and cord blood (CB) cells (A), CD33^+^ AML and peripheral blood (PB) cells (B). (**C**) Principal component analysis of the metabolic profile of CD34^+^ and CD33^+^ AML cells showing no separation between the two subgroups. (**D**) Biochemical importance plot of the top 30 metabolites contributing to group separation between CD34^+^/CD33^+^ AML and CD34^+^ cord blood/CD33^+^ peripheral blood cells. Red and blue arrows indicate increased or decreased metabolite levels in AML cells compared with CTRL cells (|fold change|≥2, *q*≤0.05), respectively. (**E**) Random Forest classification comparing CD34^+^/CD33^+^ AML and CD34^+^ cord blood/CD33^+^ peripheral blood cells.

**
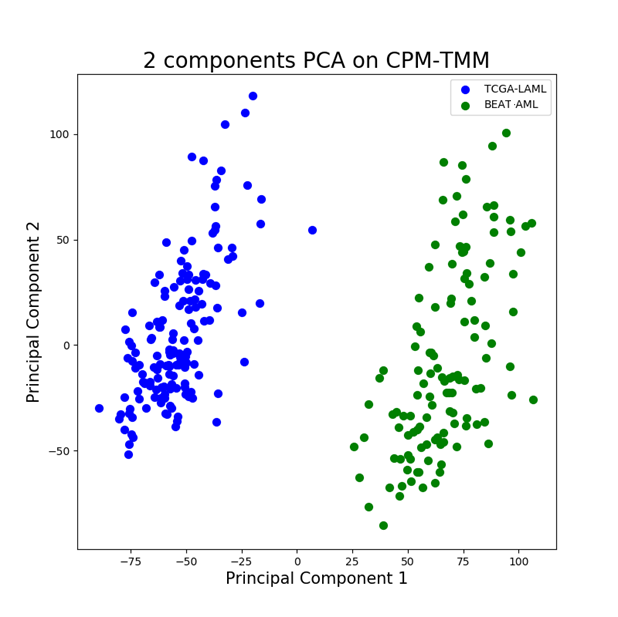
Figure S5. Two-component PCA on CPM-TMM data from the TCGA and BEAT AML cohorts.** The PCA shows a dataset-effect in sample separation.

**Figure S6. Clinical outcome and gene expression profile of *NPM1*/cohesin-mut and *NPM1*-mut AML.** (**A**) Kaplan-Meier survival curves of *NPM1*/cohesin-mut (n=31) and *NPM1*-mut (n=89). (**B**) RNA-seq data from TCGA and BEAT AML cohorts were separately analyzed and a core of differentially expressed genes (|fold change|≥2 and *p*val≤0.05 in both datasets) was defined. Data **
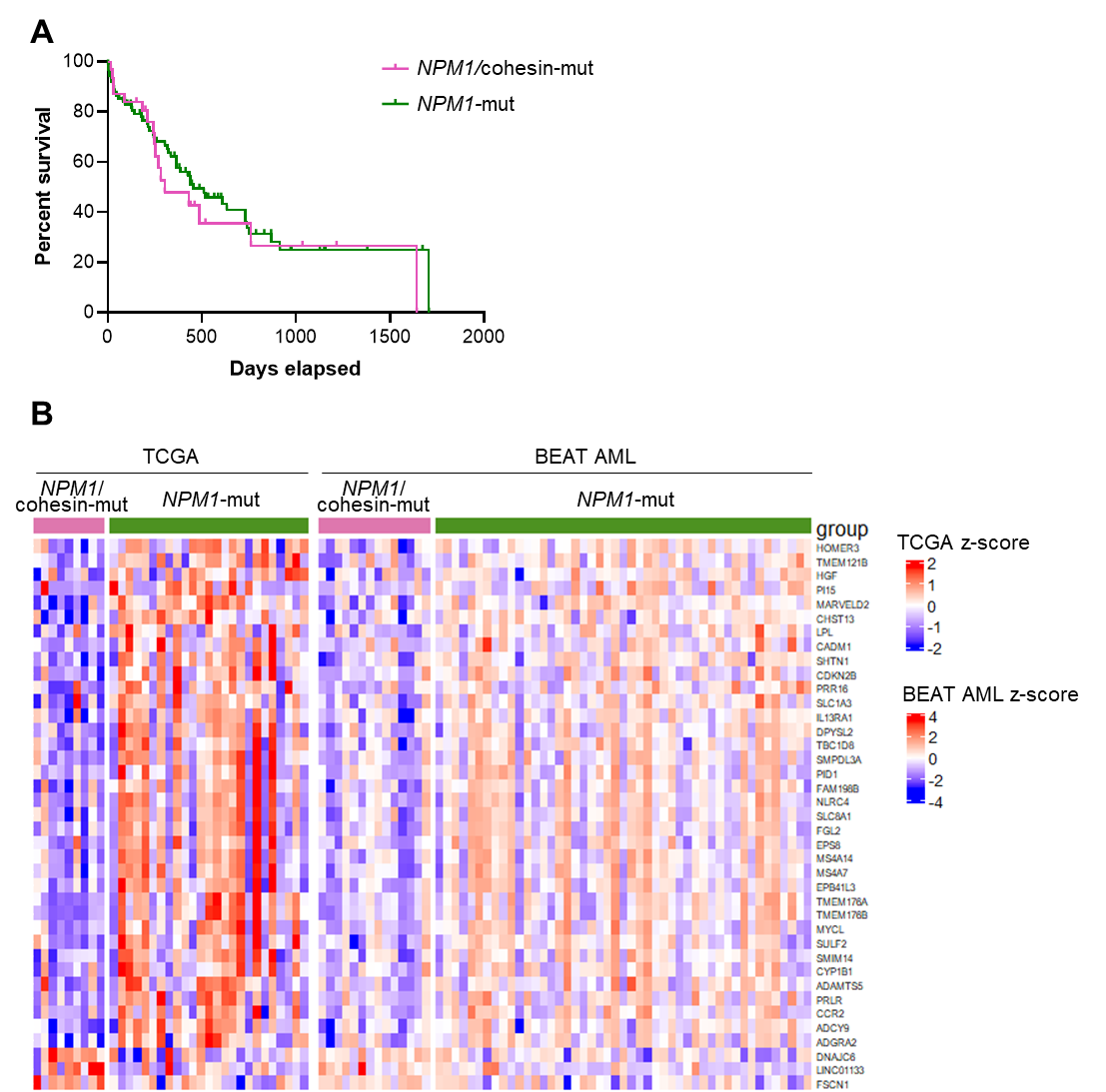
**were centred through a z-score transform. Colours were linearly interpolated according to break values and corresponding colours through CIE Lab colour space.

**
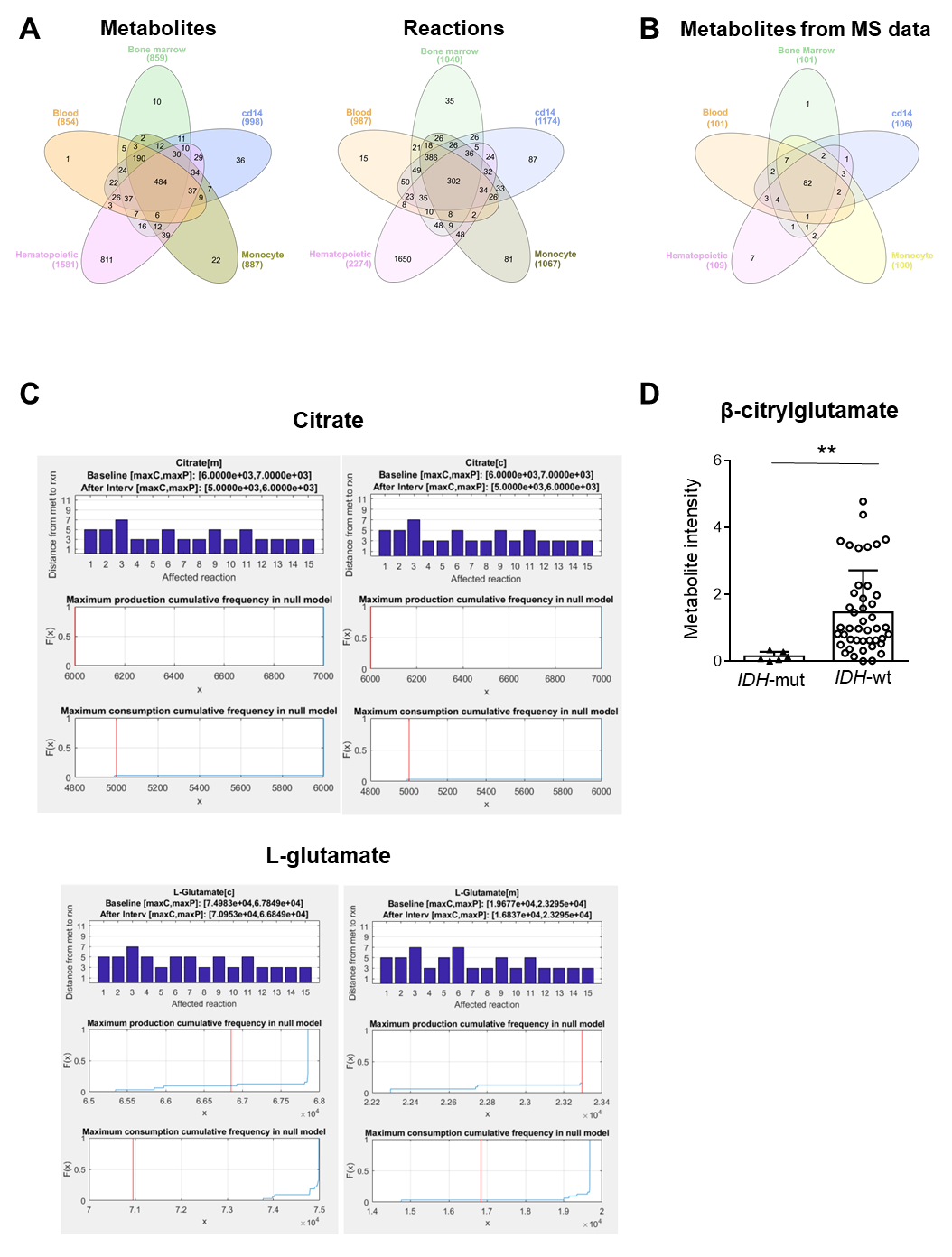
Figure S7.**

**Figure S7. Selection and validation of a metabolic model for constraint-based analysis** **by modelling the effect of *IDH* mutations.** (**A**) Overlap of metabolites and reactions annotated by mCADRE subsystems in the context-specific models: bone marrow, blood, CD14^+^ cells and monocyte and the hematopoietic MinMax model. (**B**) Mapping of metabolites from our MS profile in the models. (**C**) Output of the disruption introduced by maximizing 2-HG production and depleting α-KG on the network metabolite production and/or consumption capabilities (which simulated the effect of *IDH* mutations). Along with the distance in the stoichiometric network between the perturbed reactions and the metabolites, null models were generated (n=50), with random genetic perturbations, and the effect of the *IDH* perturbation was compared to that throughout the null models. The model predicted an alteration in L-glutamate and citrate. The results were supported by the following experimental evidence: (i) 2-HG impairs glutamate biosynthesis via transaminase inhibition [15]; (ii) inhibition of glutaminase, the enzyme that converts glutamine into glutamate, is synthetic lethal in *IDH*-mutant AML ^19^; (**D**) the level of intracellular β-citrylglutamate, the product of citrate and L-glutamate ligation by beta-citrylglutamate synthase, was significantly reduced in *IDH-*mut AML (n=6) versus *IDH*-wt (n=44). Metabolite intensity normalized on DNA concentration is shown in the plot. Significance was obtained by Welch *t*-test (***p*≤0.01).

**
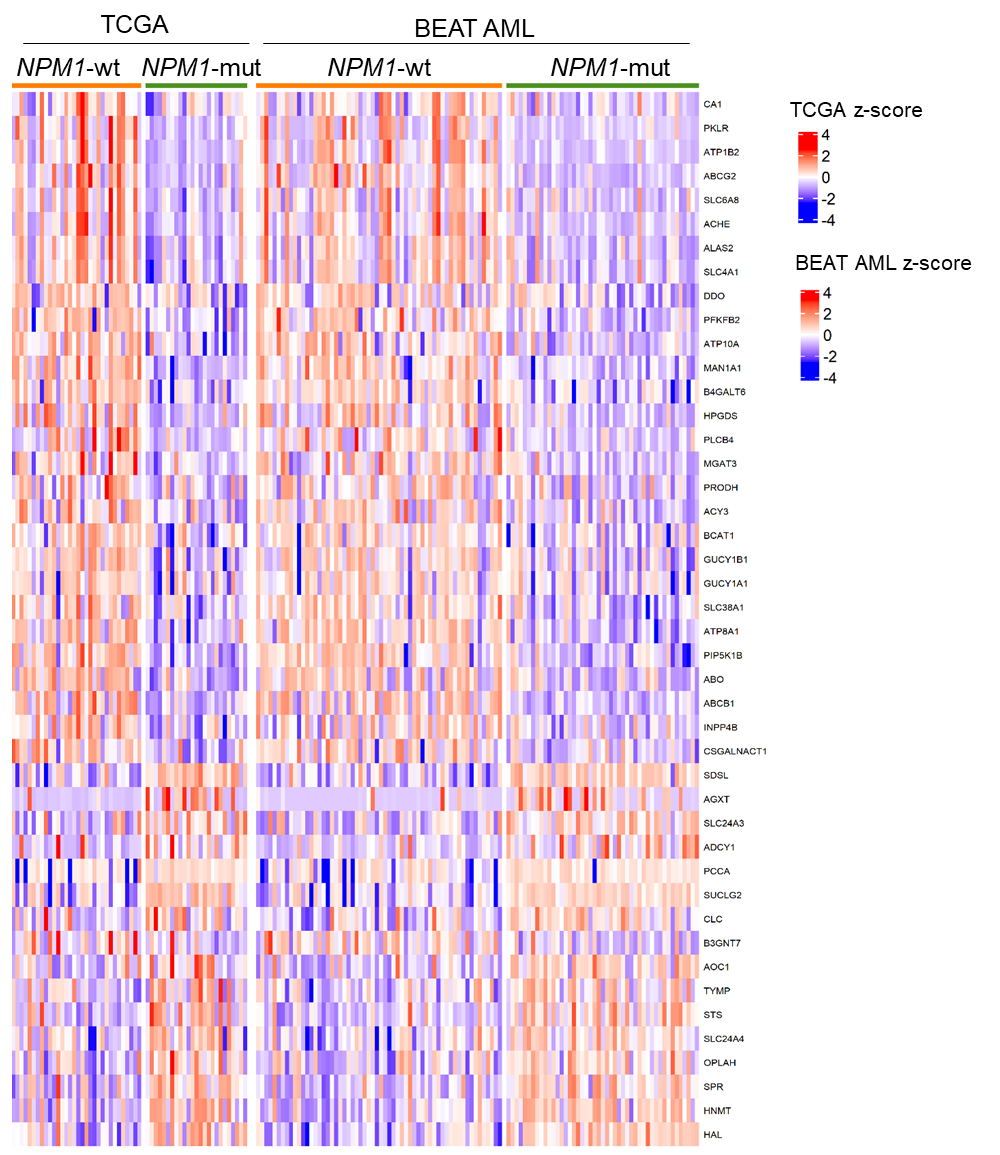
Figure S8. Metabolism-related transcriptomic differences between *NPM1*-mut and *NPM1*-wt AML.** Analysis of RNA-seq data from the TCGA (n=32 *NPM1*-wt, n=33 *NPM1*-mut) and BEAT AML (n=60 *NPM1*-wt, n=47 *NPM1*-mut) cohorts. Since *NPM1*-mut AML generally associate with normal karyotype (NK), *NPM1*-wt cases with NK were used in the analysis. Expression differences in enzyme-encoding genes are shown. Data were centred through a z-score transform. Colours were linearly interpolated according to break values and corresponding colours through CIE Lab colour space.

**Figure S9. Protein-protein interaction network of genes involved in the reactions that were specifically perturbed in *NPM1*/cohesin-mut AML** (enrichment *p*<1x10^-16^)**.
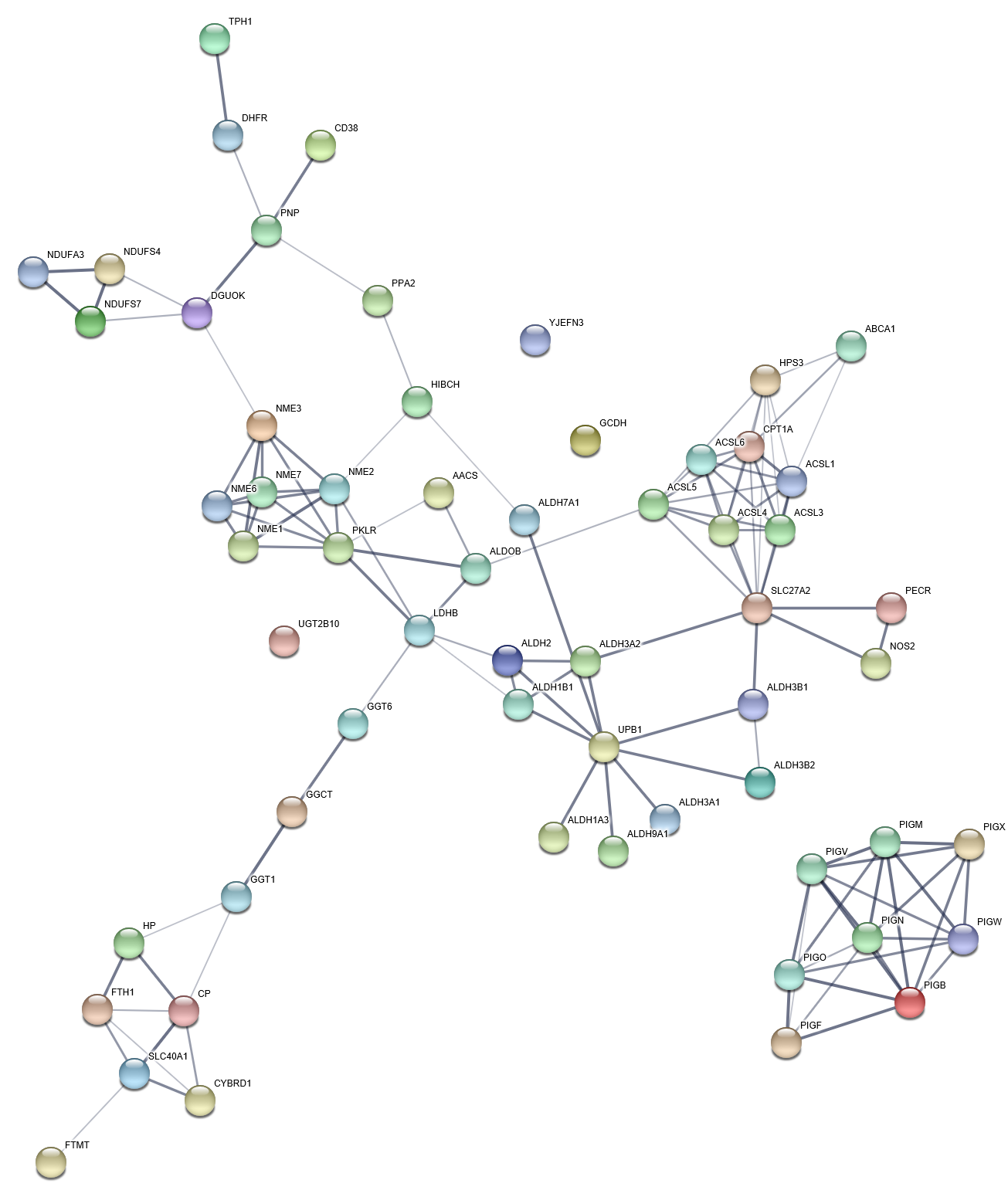
**
